# Entangled hydrophobic and hydrophilic networks in carbon monoxide dehydrogenase

**DOI:** 10.1039/d6sc04231g

**Published:** 2026-08-30

**Authors:** Shalini Yadav, Dimitrios A. Pantazis

**Affiliations:** a Max-Planck-Institut für Kohlenforschung Kaiser-Wilhelm-Platz 1 45470 Mülheim an der Ruhr Germany dimitrios.pantazis@kofo.mpg.de yadav@kofo.mpg.de

## Abstract

Carbon monoxide dehydrogenase (CODH) is a crucial enzyme involved in the global carbon cycle and fixation by catalysing the reversible interconversion of CO_2_ and CO. Although major structural features of the enzyme are known, key connections between the structure and function remain unclear. Here, we employ large-scale unconstrained all-atom molecular dynamics simulations to investigate the key catalytic features of CODH, including (i) the implications of CODH dimerization, (ii) substrate and water transport cavities, (iii) the impact of global and local hydration, and (iv) protonation-dependent local solvation around metallocofactors. The results show that dimerization involves strong interlocking near the metal clusters, with free energy analysis highlighting the role of inter-monomer hydrogen bonding. Importantly, cavity and channel analysis reveals parallel hydrophilic and hydrophobic pathways, which enable efficient CO_2_/CO and water transport to the catalytic site. This likely reflects evolutionary optimization of spatially intertwined substrate access and hydration. Enhanced sampling nearly doubled enzyme hydration relative to the crystallographic model, especially near the C-clusters, while His96, Ile567, and Ser525 rearrangements govern distinct hydration pathways around the active site. Furthermore, the protonation state of His93 controls the catalytically essential water network. Our study provides an atomistic view of CODH as a finely tuned molecular nanomachine, in which entangled hydrophilic and hydrophobic networks coordinate substrate and water delivery to the C-cluster, while key protein-matrix residues act as strategic gatekeepers. Collectively, these findings reveal how dimerization, hydration, and local protein matrix cooperatively regulate critical aspects of CODH function.

## Abstract 2

Molecular dynamics simulations reveal atomic details of spatially entangled hydrophobic and hydrophilic pathways in carbon monoxide dehydrogenase, providing insight into the channel organization and inter-metallocofactor connectivity of the enzyme.

## Graphical abstract

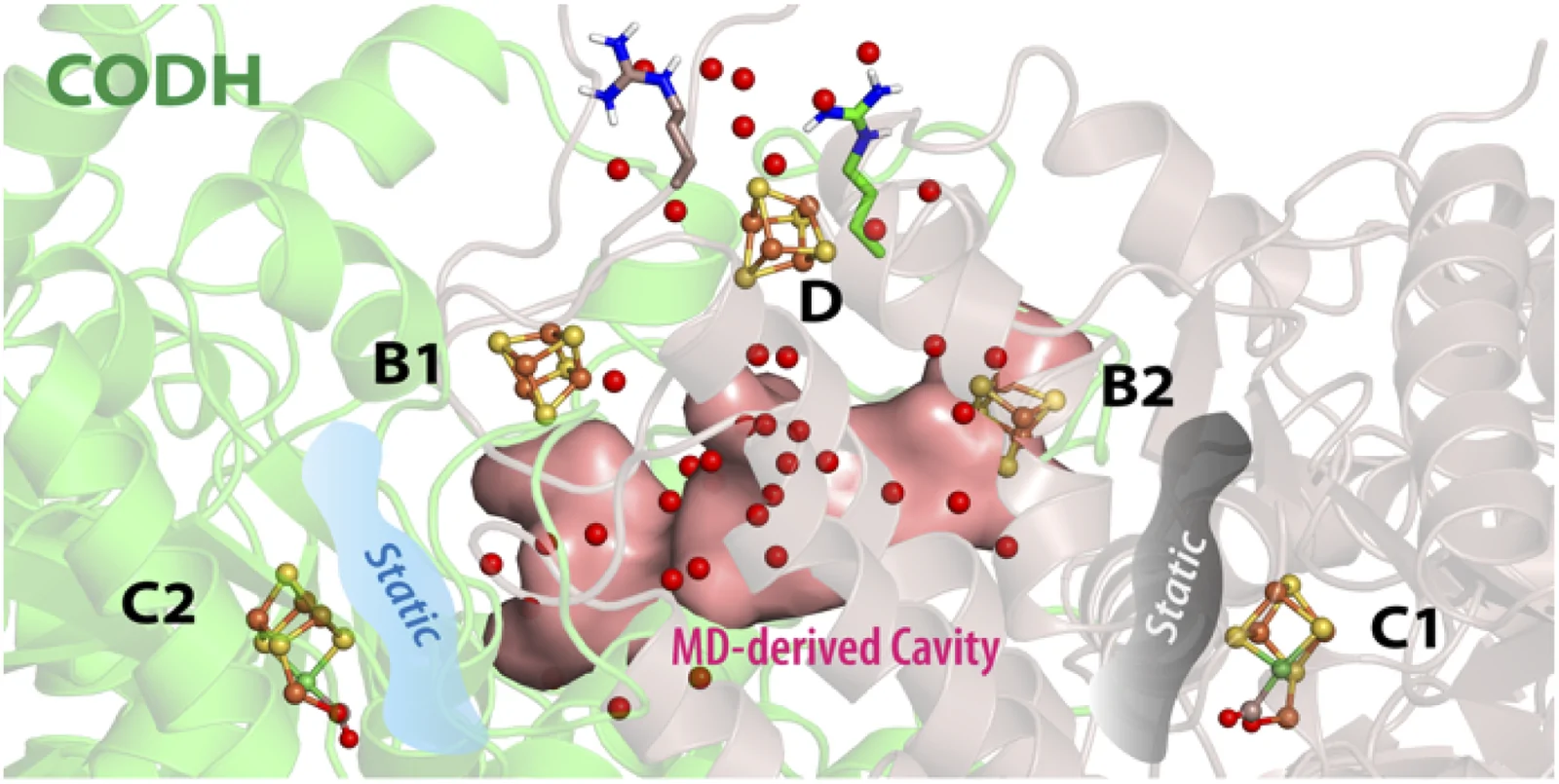

## 1. Introduction

Carbon monoxide dehydrogenase (CODH) is a homodimeric enzyme that catalyzes the interconversion of carbon monoxide (CO) and carbon dioxide (CO_2_) with remarkable efficiency, high turnover rates, and low overpotential, making it a key player in global carbon cycling and carbon fixation.^1–6^ Two types of CODH exist: the aerobic Mo–Cu-containing enzyme and the anaerobic enzyme containing a Ni–Fe cofactor. In anaerobic CODHs, the catalytic activity relies on distinct Fe–S clusters, namely, the B-cluster [Fe_4_S_4_], D-cluster [Fe_4_S_4_], and the unique nickel-incorporating C-cluster [NiFe_4_S_4_].^4,7–10^ The C-cluster serves as the catalytic center, while the B- and D-clusters form part of the electron transfer machinery, ensuring seamless flow of electrons during the reaction cycle. The efficient mechanism of CODH, and also its structural complexity, positions it as a key model system for understanding enzymatic carbon cycling and electron transfer, potentially forming the basis for bioinspired strategies.^5,11^

**Fig. 1:**
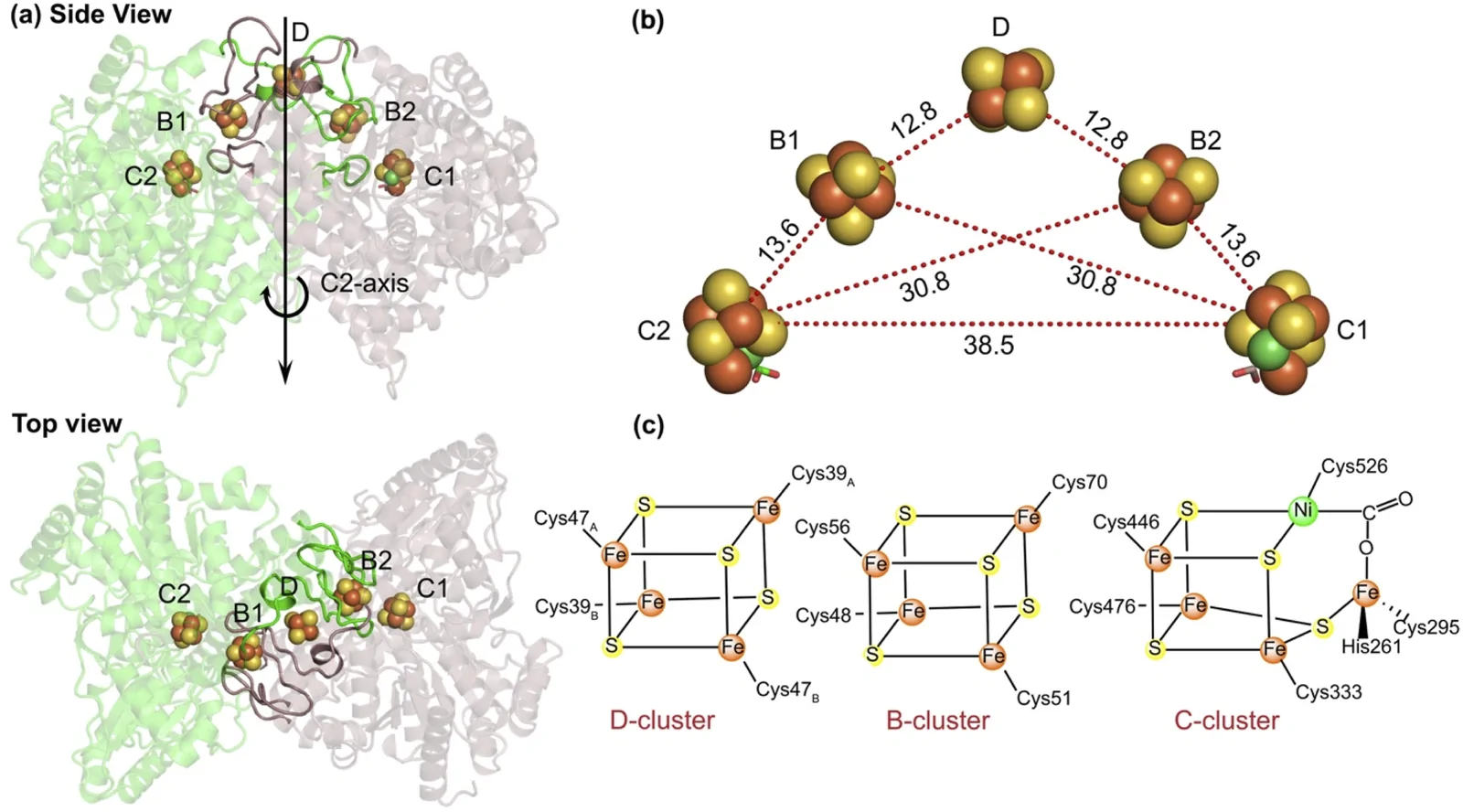
(a) Side and top view of the CODH enzyme, depicting the arrangement of clusters. (b) Distances between clusters, obtained from the crystallographic model (PDB entry 4UDX), using the center of each FeS cube as the reference point. (c) Schematic diagram of the three types of cluster in CODH.

Enzymes with oligomeric configurations, like CODH, often provide catalytic advantages by enabling the simultaneous binding of multiple substrates and enhancing overall efficiency.^12–16^ In CODH, the homodimeric architecture is characterized by five metal clusters arranged in a V-shape within the protein matrix (Fig. 1a). A visual analysis provides valuable insights into the sophisticated structural organization of CODH II from *Carboxydothermus hydrogenoformans* (CODH_CH_). As illustrated in Fig. 1b, considering just the monomeric subunit, the C-cluster (C1) and the B-cluster (B1) are spatially separated by approximately 30.8 Å (crystallographic distance), limiting their interaction due to a large separation distance. In contrast, within the dimeric assembly of CODH_CH_, the two monomeric subunits are fused in a manner that brings the B-cluster (B2) of the second subunit into close proximity (13.6 Å) with the C-cluster (C1) of the first subunit (Fig. 1b and S1). Likewise, the B-cluster (B1) of the first subunit is positioned near the C-cluster (C2) of the second subunit. The fundamental reasons for this arrangement of metallocofactors in CODH remain unclear. A deeper understanding of the connections between structural aspects of CODH and their implications is crucial for elucidating the functional principles of the catalytic mechanism as well as for understanding the evolutionary optimization of the enzyme.

An important aspect of the structural organization is the incorporation of distinct pathways that facilitate substrate entry and product release, solvent exchange, proton transfer, and ion transport.^17–22^ In CODH, structural studies have offered insights into the substrate/product (CO_2_/CO) transport pathways.^23–26^ In 2001, Dobbek *et al.* identified a continuous substrate channel in CODH_CH_, characterized by both hydrophobic and hydrophilic regions for substrate and water movement, respectively.^8^ Later, Xe-binding X-ray crystallography of the bifunctional multienzyme complex CODH/acetyl-CoA synthase (CODH/ACS) highlighted a long (138 Å) hydrophobic gas channel connecting the CODH and acetyl-CoA synthase (ACS) active sites,^27,28^ facilitating the transfer of CO from CODH to acetyl-CoA which further participates in the Wood–Ljungdahl pathway for carbon fixation. In a similar context, a study by Wang *et al.*^29^ using molecular dynamics (MD) simulations unveiled a dynamically formed gas channel in CODH/ACS that serves as the entry route for CO_2_ to the C-cluster. This transient channel, created by protein conformational fluctuations, is distinct from the structurally identified static channel. More recently, Xe crystallography of a monofunctional CODH from *Desulfovibrio vulgaris* (CODH_DV_) visualized two branched gas channels leading to the active site, with one branch showing similarity to channels in bifunctional CODH/ACS.^30^ Although X-ray crystallography has elucidated key structural features of CODH,^8–10,27^ it offers limited insight into the dynamic behavior of cavities or channels near the metal clusters. Moreover, although past studies have focused on substrate access channels in both monofunctional CODH and bifunctional CODH/ACS,^29–31^ specific pathways and mechanisms governing water movement remain poorly understood. This is particularly notable given the central role of water in the reversible interconversion of CO and CO_2_.

To address these knowledge gaps, the present study employs large-scale MD simulations to explore structural and functional aspects of CODH. Specifically, we aim to (a) elucidate structural aspects of the dimeric assembly of CODH, (b) map prominent cavities related to the metal clusters of the enzyme, (c) analyze the extent and distribution of hydration near the functionally relevant metal clusters, and (d) study the effect of the protonation state of catalytically crucial residues. MD simulations offer the unique advantage of capturing dynamic processes at the atomic level, allowing us to uncover intricate details about substrate and solvent transport pathways. By integrating these dynamic insights with existing structural data, the present study enhances our understanding of the architecture of CODH and lays the groundwork for atomic-level analysis of functional aspects in biological CO_2_/CO interconversion.

## 2. Computational details

### 2.1. System parametrization

The initial coordinates for the CODH enzyme were sourced from the crystallographic data available in the Protein Data Bank under the accession code 4UDX,^9^ (resolution: 1.03 Å, see Table S1 for a collection of available structures), pertaining to the organism *Carboxydothermus hydrogenoformans* (CODH_CH_). This CODH II was selected because it is a well-characterized enzyme for which high-resolution crystal structures are available, making it particularly suitable for structure-based MD simulations. X-ray crystallography has elucidated that CODH functions as a homodimer, with its subunits linked by a [Fe_4_S_4_] cluster (the D-cluster). Within each subunit, another [Fe_4_S_4_] cluster, termed the B-cluster, and a [NiFe_4_S_4_] cluster, known as the C-cluster, are present. It is at the C-cluster where the catalytic interconversion between CO_2_ and CO occurs.^5^ The modeled CO_2_-bound C-cluster corresponds to a reduced C_red2_–CO_2_-like state. This state was selected because C_red2_–CO_2_ is experimentally considered the most relevant and chemically appropriate CO_2_-bound state of the C-cluster.^10^ From the monomeric subunit documented for CODH_CH_ in the PDB file, we generated the complete dimer utilizing the symmetry mates tool in PyMOL. The functional significance of water molecules at the catalytic site of an enzyme is often profound, contributing significantly to its enzymatic activity. Therefore, to accurately validate the physiological context of the protein environment, all crystallographic water molecules were precisely positioned according to their respective coordinates within the crystal structure. Afterwards, each of the three metal cofactors (Fig. S2) of the CODH_CH_ enzyme underwent separate parametrization using the MCPB^32^ (Metal Center Parameter Builder) tool of the AMBER 22 package.^33^

In order to generate appropriate force field parameters for the metal cofactors, we followed an established protocol^32^ to determine bonding information and partial atomic charges from two sets of quantum mechanical (QM) calculations. These were carried out with the ORCA 6.0.^34^ program using the B3LYP^35,36^ functional and the 6-311G* basis set^37^ with the CPCM solvation scheme: (a) in the small model, only the isolated metal cluster and its coordinating residues were optimized to determine the bonding parameters between the metal and its immediate surrounding; (b) in the large model, for more accurate charge information of the metal cluster, coordinating residues were extended to include the backbone (Cα atom) of the adjacent residue from the protein matrix. The C-cluster model was treated with a total formal charge of −4 and a doublet spin multiplicity. No predefined broken-symmetry coupling scheme was imposed; the unrestricted KS-SCF calculation converged to a pair-wise antiferromagnetically coupled solution (Fe1 and Fe3 with positive spin populations of +3.30 and +3.13, Fe2 and Fe4 with negative spin populations of −3.25 and −2.91, and a nominally diamagnetic Ni site). The overlay of the crystallographic and QM-optimized geometries of the C-cluster shows that the optimized small model closely preserves the experimental CO_2_-bound C-cluster structure (Fig. S2c). The information about equilibrium bond lengths, equilibrium angles, bond force constants and angle force constants was derived from the Hessian matrix of QM optimized geometries with a modified Seminario approach,^38^ which was further incorporated into the *frcmod* file. Afterwards, we employed the RESP^39,40^ (Restrained Electrostatic Potential) charge fitting method with the Multiwfn^41^ program to obtain the partial electrostatic atomic charges for the cofactors from the QM optimized geometries.

**Fig. 2:**
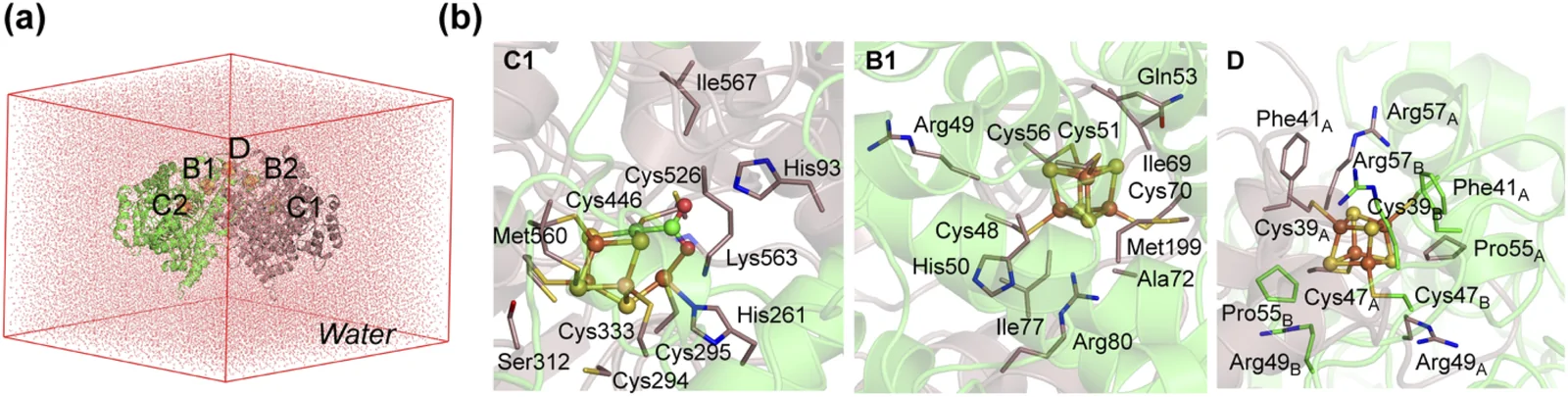
(a) Illustration of the molecular mechanics model of the CODH_CH_ dimer, showcasing the enzyme with solvation by water molecules. The simulation box dimensions are 134.995 × 108.640 × 140.127 Å^3^. (b) Spatial organization of amino acid residues surrounding the C1-cluster, B1-cluster and D-cluster within a 4 Å radius, depicting the immediate biochemical environment. Note that the C2-cluster and B2-cluster have the same immediate environment in subunit-2.

After successful parametrization of the individual cofactors, their parameters were combined to create a unified CODH_CH_ system setup. Protonation states of titratable amino acids (Asp, Glu, and His) proximal to the cofactors were assigned based on their p*K*_a_ values calculated with the Propka 3.0 (ref. 42) module and on our own analysis of local hydrogen-bonded networks. Specifically, the p*K*_a_ prediction was used as the first criterion, but the final protonation state was not assigned solely from the calculated p*K*_a_ value. The microenvironment of each titratable residue was further inspected in terms of the hydrogen-bonding pattern, distance and orientation of nearby donor/acceptor groups, solvent accessibility, proximity to charged residues, and possible stabilization of the protonated or deprotonated form. When the p*K*_a_ prediction was inconsistent with the observed hydrogen-bonding network, we prioritized the chemically reasonable protonation state that best preserved the local interaction pattern and avoided unrealistic donor–donor or acceptor–acceptor contacts. For example, cysteine residues directly coordinated to the metal clusters were kept in the deprotonated thiolate form, irrespective of their predicted p*K*_a_ values, to get a reasonable cluster geometry. Similarly, for metal-proximal histidines, the HID/HIE assignment was made by examining which imidazole nitrogen was oriented toward the metal site and which nitrogen was available for hydrogen bonding with the surrounding protein or solvent. When both nitrogens could contribute to stabilizing interactions in a polar environment, the doubly protonated HIP form was also considered. The most probable protonation state was therefore selected by combining (i) p*K*_a_ prediction, (ii) local hydrogen-bonding compatibility, (iii) electrostatic stabilization by neighboring residues or cofactors, and (iv) chemically reasonable coordination with nearby metal centers or cofactors. This combined approach allowed us to avoid relying solely on automated p*K*_a_ values and ensured that the assigned protonation states were chemically reasonable within the local protein environment. Missing hydrogens were added using the tLEaP module within the AMBER22 package, utilizing the FF19SB^43^ force field. The resulting system was solvated in a rectangular box using the TIP3P^44^ water model, extending up to 15 Å from the protein surface. To maintain electrical neutrality, 13 Na^+^ ions were included in the system (Fig. 2).

### 2.2. Classical molecular dynamics simulations

After appropriate parameterization, the system was minimized to eliminate unfavorable contacts and achieve geometric relaxation. This minimization procedure consisted of 5000 steps using the steepest descent method, followed by 5000 steps utilizing a conjugate gradient approach. Subsequently, the system underwent gentle annealing for 50 ps under the NVT ensemble, with constraints applied to the protein to adjust the temperature. Next, density equilibration was conducted for 1 ns under the NPT ensemble, maintaining a constant temperature of 300 K and pressure of 1.0 atm. Temperature and pressure were controlled using the Langevin thermostat^45^ with a collision frequency of 2 ps^−1^ and the Berendsen barostat^46^ with a pressure relaxation time of 1 ps. The 1 ns density equilibration phase involved an MD simulation with weak restraints under periodic boundary conditions (PBCs),^47^ continuing until the system achieved uniform density. Once uniform density was attained, all previously applied restraints (during heating and density equilibration) were removed. The system was subsequently subjected to a further 10 ns of equilibration followed by a production MD simulation. To improve configurational sampling and assess reproducibility, three independent 200 ns trajectories were generated, corresponding to a total simulation time of 600 ns. Each 200 ns trajectory was propagated as four consecutive 50 ns production segments, with each segment restarted from the endpoint of the previous one. The four segments therefore together constitute one continuous 200 ns trajectory for analysis. The simulation utilized the SHAKE^48^ algorithm to constrain hydrogen atoms. Long-range electrostatic interactions were treated using the particle mesh Ewald (PME)^49^ method. Simulation was performed using the GPU version of the AMBER22 package.^50,51^ Analysis of the resulting trajectory was conducted using the CPPTRAJ^52^ module of the AMBER package. Visualization of the trajectories was carried out using VMD,^53^ while figures were prepared with PyMOL.^54^ To accurately reflect the statistically most significant enzyme geometry during MD sampling, we extracted the most populated snapshot by using the *k*-means approach implemented in CPPTRAJ and used that for figure preparation.

### 2.3. MM/GBSA calculations

To enhance the efficiency of atomistic simulations, implicit solvent models approximate the surrounding solvent as a dielectric continuum, significantly reducing the system size by eliminating the need to explicitly model individual solvent molecules.^55^ This simplification not only decreases computational cost but also accelerates conformational sampling.^56–60^ Among these models, the Generalized Born (GB) approach is one of the most widely adopted, particularly in combination with AMBER force fields for protein systems.^61,62^ A common application of this method is the MM/GBSA (Molecular Mechanics/Generalized Born Surface Area) framework, which combines molecular mechanics energies with solvation free energy estimates derived from the GB model and solvent-accessible surface area calculations. In this study, MM/GBSA calculations were performed using the MMPBSA.py^63^ module in the AMBER simulation suite to investigate the energetics of subunit interactions within the dimeric protein. Simulations were conducted on the protein-only geometry, excluding water and ions, with dielectric constants set to 1 for the solute and 80 for the solvent to mimic physiological conditions.

## 3. Results and discussion

### 3.1. Structural stability of dimeric CODH

**Fig. 3:**
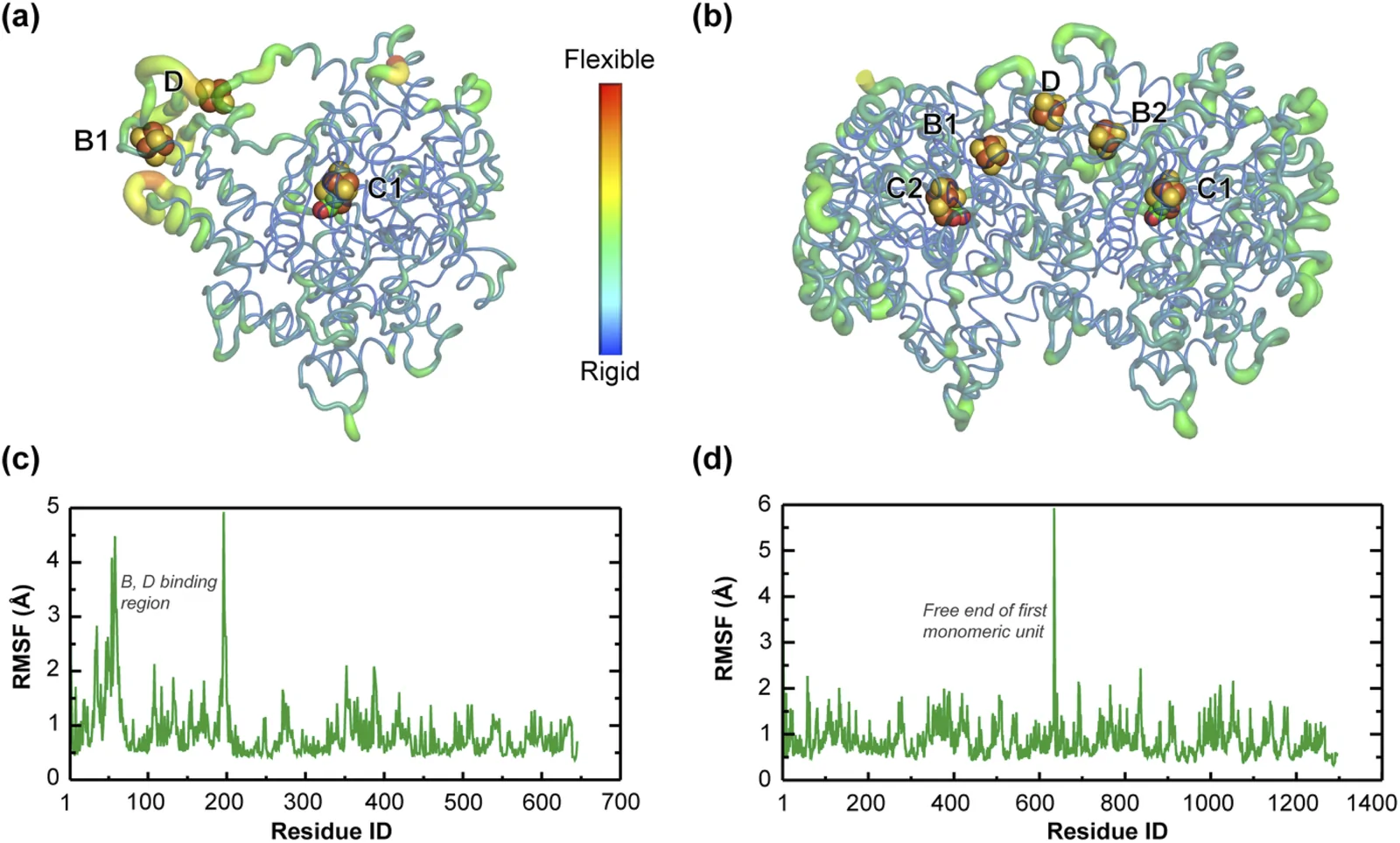
Structural visualization highlighting regions of significant conformational fluctuations observed in (a) monomeric and (b) dimeric forms of CODH_CH_, with corresponding RMSF profiles (c and d) derived from their respective MD simulation trajectories.

Understanding the influence of dynamics on protein matrices is crucial for elucidating the structural and functional implications of oligomerization in enzymatic assemblies. To investigate the impact of dynamics on the monomeric and dimeric assemblies of CODH_CH_, we conducted independent 200 ns molecular dynamics (MD) simulations (Fig. 3 and S3). The simulations provided valuable insights into the structural stability and behavior of these systems. As illustrated in Fig. S3c, the RMSD profile of the dimeric assembly indicates good structural stability, reflected by a stable protein RMSD throughout the simulation. An isolated monomeric subunit undergoes much more significant conformational fluctuations during the simulation compared to the dimeric assembly (Fig. 3a and b). Major fluctuations predominantly occur in regions surrounding the B-cluster and D-cluster within the monomeric units. In contrast, the interlocked dimeric unit, characterized by its compact and closely packed architecture, experiences limited structural fluctuations. This arrangement stabilizes the arrangement of metal clusters and minimizes structural perturbations (Fig. 3c and d), promoting efficient functioning of the enzyme.

#### 3.1.1. Inter-subunit interaction stabilizes the B- and D-clusters

The primary differences between the monomeric and dimeric forms of CODH_CH_ were observed near the binding regions of the B and D-clusters. The catalytic C-cluster remains largely unaffected by the large fluctuations due to its buried site inside the protein matrix. To further investigate the B- and D-cluster regions, we conducted a comprehensive analysis to map the interactions within the homodimeric CODH_CH_, identifying key residues that stabilize the complex. Our analysis categorized these residues into covalently ligated residues and nearby hydrogen-bonded residues critical for maintaining cluster stability.

**Fig. 4:**
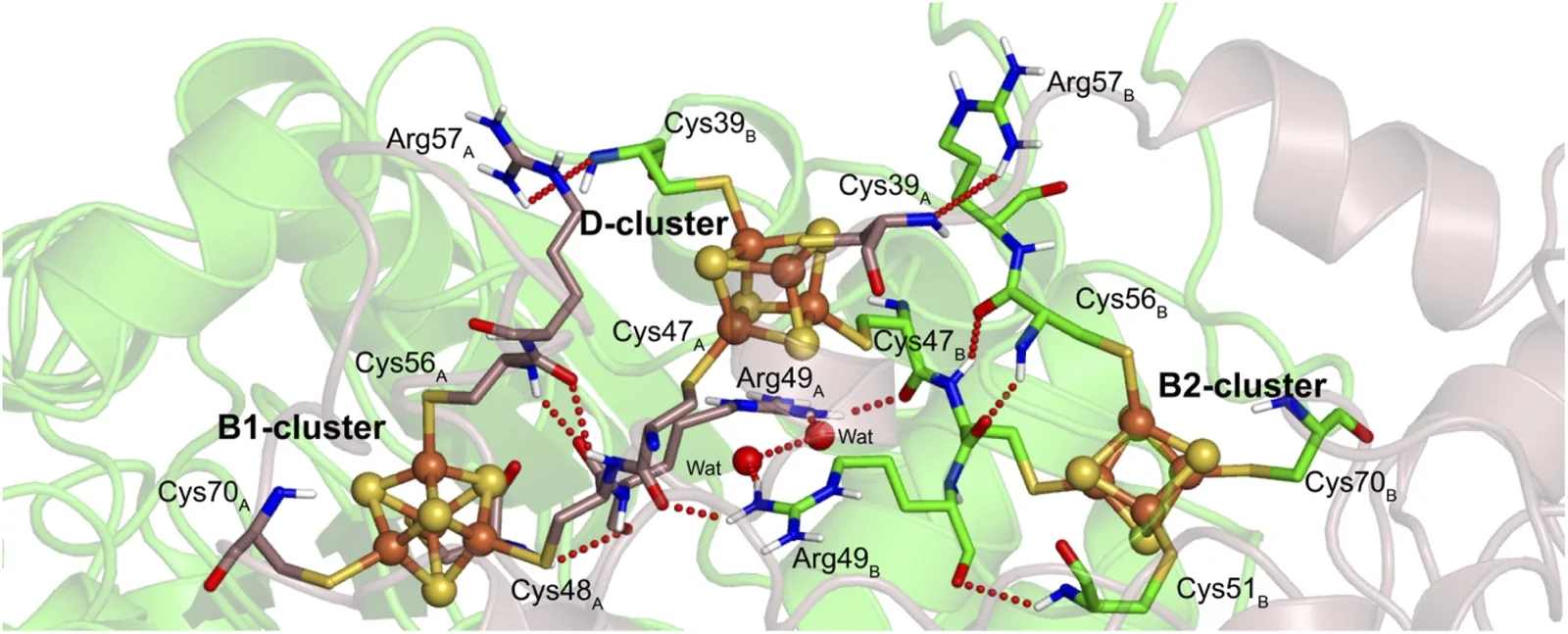
Interactions between amino acid residues surrounding the B- and D-cluster regions in the homodimeric unit. Brown represents residues that belong to subunit-A and green represents residues from subunit-B.

The monomeric form of CODH_CH_ contains relatively few interacting residues around the clusters (Fig. S4). However, dimerization embeds the cluster within an extensive interaction network. Notably, Arg49_B_ plays a pivotal role in the dimeric assembly by interacting with Arg49_A_ and two water molecules, forming a cradle-like structure that accommodates the D-cluster. Note that subscripts A and B are used to indicate residues from subunits A and B. Furthermore, inter-subunit interactions between residue pairs Arg57_A_–Cys39_B_, Cys47_A_–Arg49_B_, Arg49_A_–Arg49_B_, Cys39_A_–Arg57_B_, and Arg49_A_–Cys47_B_, significantly enhance the hydrogen-bonding network (Fig. 4). These interactions reinforce the structural integrity of the B- and D-clusters and facilitate the formation of a more stable geometric arrangement, thereby optimizing the electron transfer machinery within the enzyme. The interactions between the D-cluster and the protein matrix between subunit A and B, coupled with formation of new inter-residue and water-mediated hydrogen bonds, underscore the critical role of dimerization in stabilizing the enzymatic cofactors. In summary, the biologically assembled dimeric unit is evolutionarily optimized to enhance structural integrity and reduce fluctuations by incorporating inter-subunit H-bonding interactions, ensuring a robust and efficient electron transfer network to the C-cluster, which is critical for the enzyme's catalytic activity.

#### 3.1.2. Interfacial residue interaction energies and dimer stability

Following the above discussion, to quantitatively elucidate the molecular interactions that contribute to the enhanced stability of the dimeric assembly, we employed thermodynamic MM/GBSA calculations. Our investigation aimed to identify the critical residues at the interface of the *C*_2_-symmetric fused subunits by analyzing the total interaction energies of the protein subunits. We began with MD simulations of the enzyme, followed by detailed free energy calculations. Although the dimeric complex is naturally expected to be more stable than the monomeric form, the main objective of the residue-wise MM/GBSA decomposition analysis was to identify critical interfacial residues that underpin this stabilization (Tables S2–S4).

**Fig. 5:**
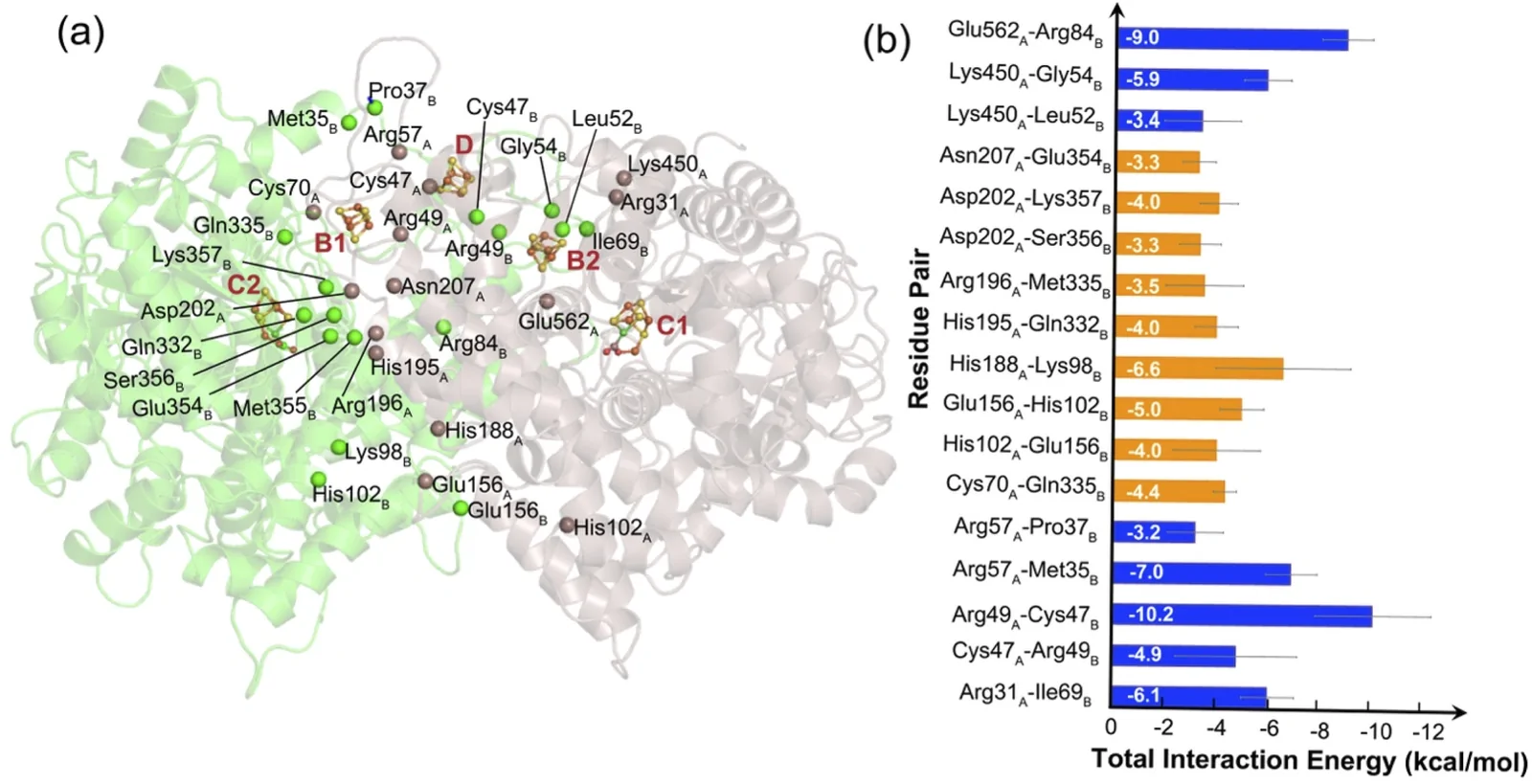
(a) Positioning of interfacial residues involved in interactions between subunit-A and subunit-B. (b) Interaction energies of interfacial residue pairs involved in the dimeric assembly. Interactions depicted in blue correspond to those observed in the B- and D-cluster regions, while interactions shown in orange represent those near the C-cluster region and beyond. The residue pairs are arranged from bottom to top in ascending order of residue number in subunit-A.

**Fig. 6:**
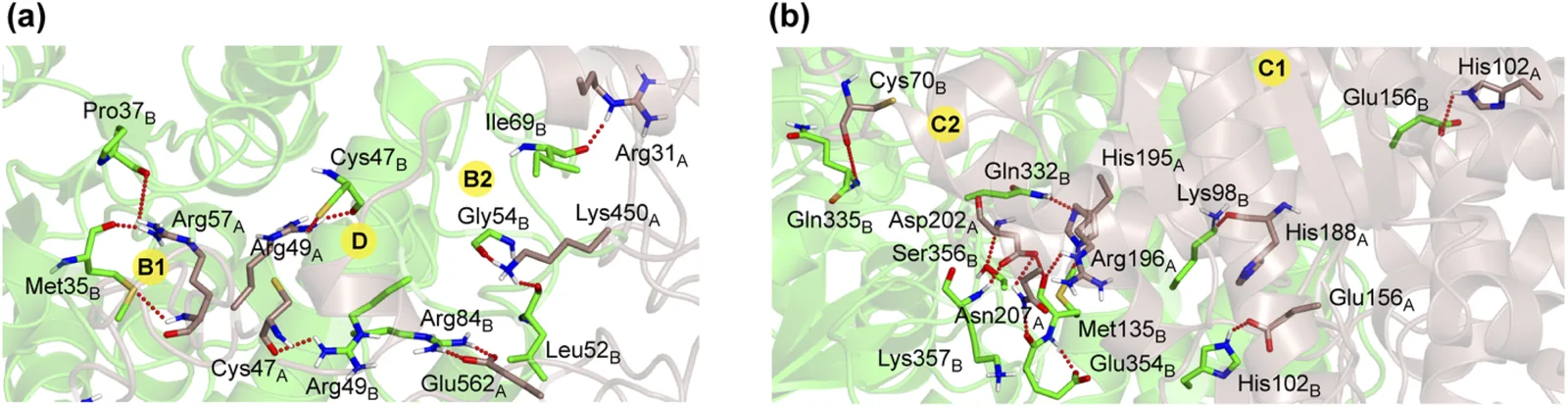
(a) Interfacial residue pairs located near the B- and D-clusters; (b) residue pairs surrounding the C1- and C2-clusters as well as pairs distributed beyond these regions.

To simplify the analysis of the interfacial interaction in CODH, we extracted only those residue pairs from residue-wise decomposition data, in which one residue from subunit A interacts with another residue from subunit B (Fig. 5a). This allowed us to identify key residue pairs that contribute most significantly to stabilization of the dimer interface (Fig. 5b). For clarity, specific residue–residue interactions are also shown in Fig. 6.

The B and D-cluster regions feature residue pairs including key arginine residues, consistent with those identified above, where their interaction was proposed to contribute to cluster stabilization. Notably, the guanidinium side chain of Arg49_A_ interacts with the thiolate and carbonyl oxygen of Cys47_B_, contributing −10.2 kcal mol^−1^ to dimer stabilization. Similarly, a salt bridge between Glu562_A_ and Arg84_B_ contributes −9.0 kcal mol^−1^. Other significant interactions include Arg31_A_ with Ile69_B_ (−6.1 kcal mol^−1^), Cys47_A_ with Arg49_B_ (−4.9 kcal mol^−1^), and dual interactions of Arg57_A_ with Met35_B_ (−7.0 kcal mol^−1^) and Pro37_B_ (−3.2 kcal mol^−1^). The histogram in Fig. 5b shows that residues associated with the B- and D-cluster regions are key contributors to stabilization compared to those in the remote matrix. Conversely, residues in the C-cluster region and beyond, contribute less to overall stabilization. This highlights the differential impact of specific residues near metallocofactors on dimer stability. Overall, our analysis points to specific residues situated near the metal clusters as well as at the interface as important for stabilizing the dimeric assembly, suggesting targets for future research into the functional roles of these interactions.

### 3.2. Functional cavities in CODH: water and substrate transport pathways

An important question in the context of CODH function is how the protein assembly and particularly the interfacial residues that contribute to dimer stabilization, also contribute to organize the pathways for the transport of essential species such as the substrate and water to the active site. Previous structural and computational studies have discussed various plausible CO_2_/CO (substrate/product) transport pathways in CODH and CODH/ACS, including both static and dynamic channels using substrate diffusion techniques.^29–31,64^ A continuous substrate channel with hydrophobic and hydrophilic regions was first identified in CODH_CH_.^8^ In CODH/ACS, a 138 Å gas tunnel connecting the C-cluster and A-clusters was mapped using Xe-binding crystallography, with specific residues shown to influence acetyl-CoA production.^27,65^ Molecular dynamics later uncovered a transient CO_2_ entry channel regulated by key histidine residues (His113/116 analogous to His93/His96 in the crystal structure) and flanked by dynamic cavities.^29,31^ Additional static cavities and forked gas channels have been experimentally visualized in monofunctional CODH_DV_, with hydrophobic and polar residues shaping these routes.^30,66^ Comparative analyses also suggest partial overlap and divergence in channel architecture between monofunctional and bifunctional CODH enzymes, with distinct residues potentially modulating access. Although previous studies have provided valuable insights into CODH, structural investigations have mostly provided static pictures of individual cavities from crystallography.^7,8,10,30^ Meanwhile, dynamic analyses have mostly focused on the immediate catalytic environment, with a focus on the C-cluster.^29,31^ Considering that all metal clusters are functionally important, mapping the cavity architecture in greater detail is essential to understand how the overall enzyme framework supports and regulates CO_2_/CO and water pathways.

Recognizing the complexity of small-molecule diffusion in proteins, it is imperative to accurately model the polar, hydrogen-bonding, and hydrophobic interactions between protein residues, solvent molecules, and the diffusing entities. To this end, using long timescale MD simulations, we identified and characterized cavities using the *fpocket* software^67^ (Text S1). To achieve precision in cavity identification, we analyzed a 200 ns MD simulation trajectory comprising 20 000 frames to construct a representative time-averaged protein structure. This strategy ensured a statistically robust and well-sampled depiction of cavities. Employing the “*mdpocket*” module of the *fpocket* software, we identified all potential cavities across the extensive and intricate CODH structure (Table S5). However, recognizing the functional significance of metal cluster regions, our analysis was refined to focus on cavities proximal to these catalytic centers.

**Fig. 7:**
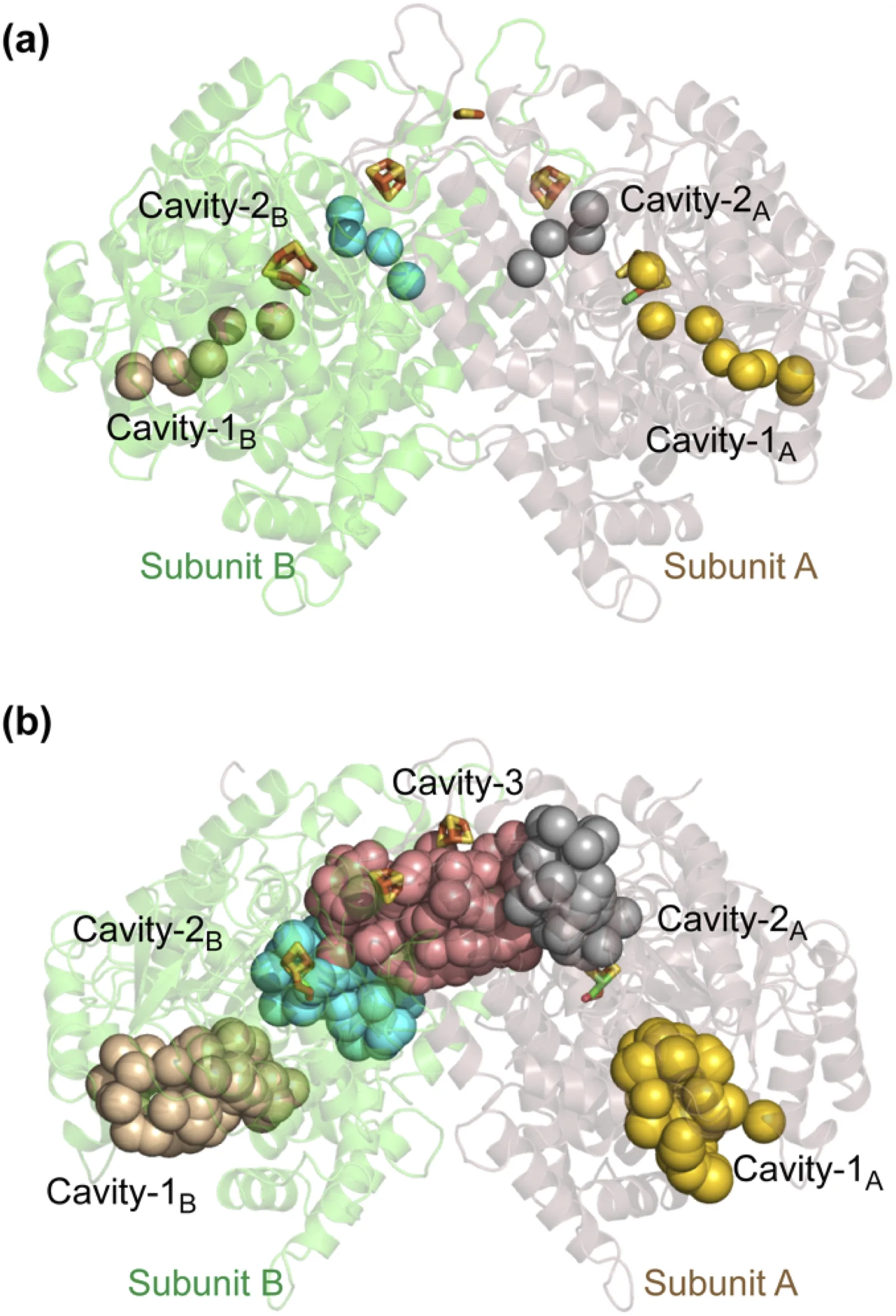
(a) Experimentally observed cavities in CODH_DV_ using Xe diffusion.^30^ (b) Depiction of key cavities observed in MD simulation. Note that CODH_DV_ and CODH_CH_ exhibit a highly similar overall structure, differing by only ∼1.2 Å in RMSD.

As an experimental reference for the MD-derived cavities, we considered the Xe binding sites previously reported for CODH_DV_.^30^ The study exploited Xe as a surrogate probe for gaseous substrates, because Xe can populate buried hydrophobic pockets and transient voids relevant to gas transport. The crystallographic analysis identified two such cavity regions in each subunit, denoted as Cavitiy-1_A_ and 2_A_ in subunit-A, with the corresponding symmetry-related features, Cavity-1_B_ and 2_B_, present in subunit-B (Fig. 7a). Although CODH is described crystallographically as a *C*_2_ symmetric homodimer, our MD simulations reveal a slight asymmetry in cavity formation between the two subunits (Fig. 7b). This deviation likely arises from intrinsic conformational flexibility and differential residue dynamics. Factors such as local side-chain rearrangements, asymmetric interaction between residues, and solvent interactions contribute to distinct cavity geometries in each protein subunit.

The present analysis is aimed at cavity characterization and does not directly simulate substrate diffusion within the enzyme; therefore a one-to-one replication of experimentally observed pathways is not expected. Nevertheless, there is a significant degree of correspondence as internal cavities define the steric and physicochemical landscape through which small molecules may migrate. In the MD structures, cavities related to the crystallographic observations were identified in both subunits, namely Cavity-1_A_ and Cavity-2_A_ in subunit-A and Cavity-1_B_ and Cavity-2_B_ in subunit B, together with an additional cavity, termed Cavity-3. Cavity-3 is reported here for the first time and emerges dynamically in the simulation ensemble (Fig. 7). Its positioning along the metal-cluster region of CODH suggests that it may contribute to the internal connectivity around the catalytic C-cluster and neighboring metal clusters. Importantly, these analogous cavities appear more clearly in the MD structures than in the crystal structures, both in terms of their geometry and their relation to the catalytic sites, suggesting that conformational fluctuations significantly modulate internal void space. Only cavities relevant to the metal-cluster region were retained for analysis, including both internal cavities and bulk-connected cavities that may allow temporary water or substrate entry. Broad surface-exposed regions without an obvious connection to the cluster region were excluded. The final selection was based on visual inspection.

#### 3.2.1. Dual functional nature of Cavity-1_A_ and Cavity-1_B_

**Fig. 8:**
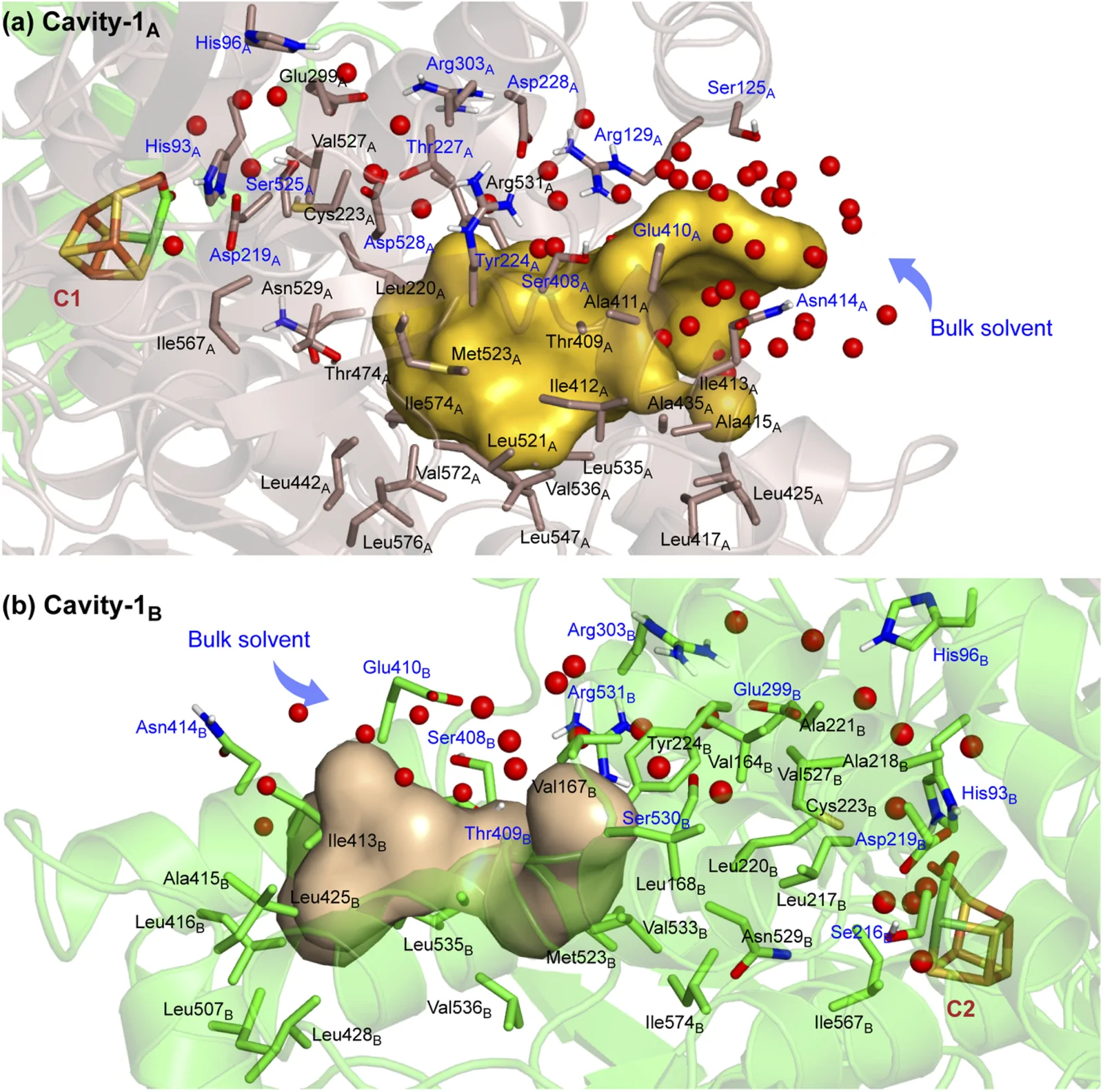
Structural representation of parallel hydrophobic (Cavity-1_A_ and 1_B_) and hydrophilic cavities in CODH, both converging at the catalytic C-cluster. Residues contributing to water flow are labeled in blue, and residues involved in the hydrophobic cavity are labeled in black.

Our MD simulations reveal that Cavity-1_A_ and Cavity-1_B_ form two analogous, surface-connected cavities that project toward the C-cluster. Compared with the static crystal structure,^30^ these are much more clearly and distinctly resolved in the MD ensemble, particularly in terms of their continuity, increased enclosure towards metal clusters, and geometric definition, indicating that the catalytically relevant internal topology of CODH is strongly shaped by dynamics that might be impossible to capture in a crystallographic snapshot. Because the two cavities occupy corresponding positions in the two subunits, their architectures are also broadly conserved (Fig. 8). In subunit A, Cavity-1_A_ is defined predominantly by a hydrophobic lining composed of Leu425_A_, Ala415_A_, Ile413_A_, Ala435_A_, Leu417_A_, Leu547_A_, Met523_A_, Val536_A_, Leu535_A_, Ile412_A_, Ala411_A_, Leu521_A_, Val572_A_, Ile574_A_, Leu576_A_, and Leu442_A_, forming an extended nonpolar pocket. This cavity then narrows toward the C-cluster through Leu220_A_, Asn529_A_, Thr474_A_, and Ile567_A_. In subunit B, the analogous Cavity-1_B_ is again built largely by hydrophobic residues, including Ala415_B_, Leu416_B_, Leu507_B_, Leu425_B_, Ile413_B_, Leu535_B_, and Met523_B_ and continues toward the catalytic region through Val533_B_, Leu168_B_, Leu220_B_, Leu217_B_, Asn529_B_, Ile574_B_, and Ile567_B_. The asymmetry in the cavity shapes seen here primarily reflects dynamic reshaping of the cavity walls by local residue motions rather than a fundamentally different cavity architecture. Thus, in both subunits, the internal route is embedded within a hydrophobic protein matrix, consistent with a pathway optimized for the movement of small gaseous substrates such as CO and CO_2_ rather than bulk solvent.

An important feature is the appearance of Ile567 at the cluster-proximal end of both cavities. Its conserved placement suggests that this residue may define the final steric contour of the substrate approach channel immediately before the catalytic center. This interpretation is crucial because Ile567 is highly conserved among CODHs, and perturbations at this position have been associated with altered substrate accessibility and catalytic behavior.^7^ In this context, Ile567 is well positioned to function not merely as a passive structural residue, but as a local gatekeeper that helps regulate the final entry of the substrate into the C-cluster environment.

A particularly noteworthy outcome of our analysis is the identification of a water-flow pathway running *in parallel* to the hydrophobic substrate cavity toward the buried C-cluster. This is important because gas-transport routes are usually expected to remain predominantly hydrophobic, while water access is often limited to prevent unnecessary solvent intrusion. In CODH, however, these two demands appear to be resolved through a dual-pathway architecture: a hydrophobic route suited for CO/CO_2_ transport and a neighboring polar route that guides water to the same catalytic region. For Cavity-1_A_, this water pathway begins near Asn414_A_ and Glu410_A_, then proceeds through Ser125_A_, Arg129_A_, Ser408_A_, Arg531_A_, Asp228_A_, Thr227_A_, Arg303_A_, Asp528_A_, and Glu299_A_, before reaching the C-cluster through His96_A_, His93_A_, and Asp219_A_. For Cavity-1_B_, the parallel water flow is again initiated by Asn414_B_ and Glu410_B_ and continues through Ser408_B_, Thr409_B_, Arg531_B_, Arg303_B_, Ser530_B_, and Glu299_B_, finally converging at the same cluster-facing residues, His96_B_, His93_B_, and Asp219_B_. The involvement of His93 and His96 in both cavities is particularly noteworthy, as these residues appear to form the final water-delivery segment to the reaction center and have also been implicated in earlier experimental and computational studies.^7,29,31,64^ This dual-channel system reflects a highly evolved enzymatic architecture to efficiently deliver CO_2_/CO and water to the active site. Overall, this parallel water flow highlights a functionally important feature of CODH: spatially proximal and even intertwined hydrophobic/hydrophilic systems that enable selective gaseous substrate transport while maintaining controlled solvent access to the buried active site.

#### 3.2.2. Cavity-2_A_ and Cavity-2_B_ systems

The Cavity-2_A_ and Cavity-2_B_ systems are relatively shorter than other identified cavities. Both cavities are located between the B- and C-clusters of the two enzyme subunits, and their high hydrophobicity scores (45.77 for Cavity-4 and 51.84 for Cavity-5) and low polarity scores (4 for Cavity-4 and 3 for Cavity-5) (Table S5) reflect their predominantly hydrophobic nature, aligning with their likely ability to act as transport pathways for CO_2_ or CO.

**Fig. 9:**
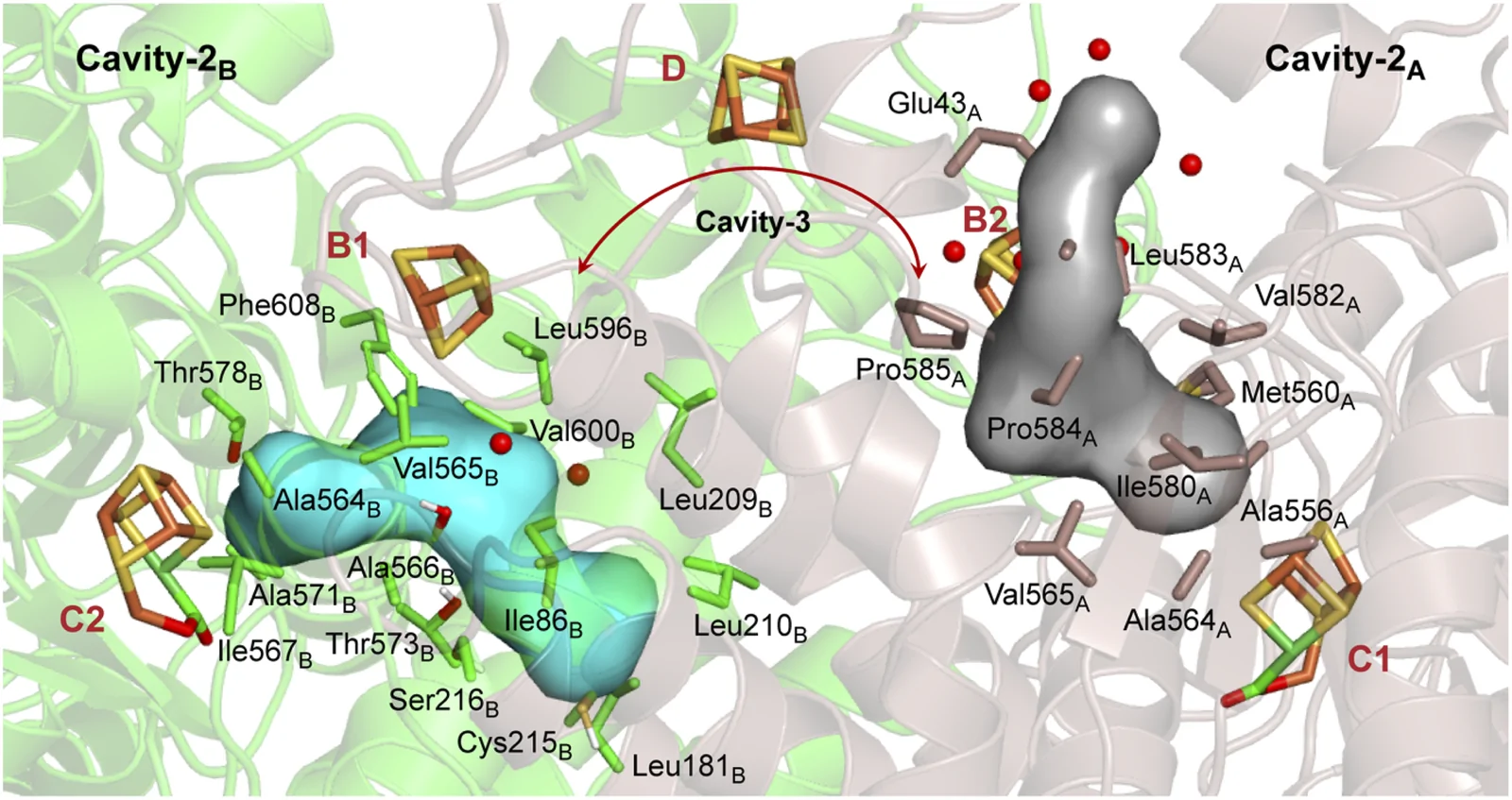
Cavity-2_A_ and Cavity-2_B_ with their surrounding residues, illustrating their roles in connecting the B1-cluster to the C2-cluster and the B2-cluster to the C1-cluster, respectively. The red arrow region represents the area covered by Cavity-3.

**Fig. 10:**
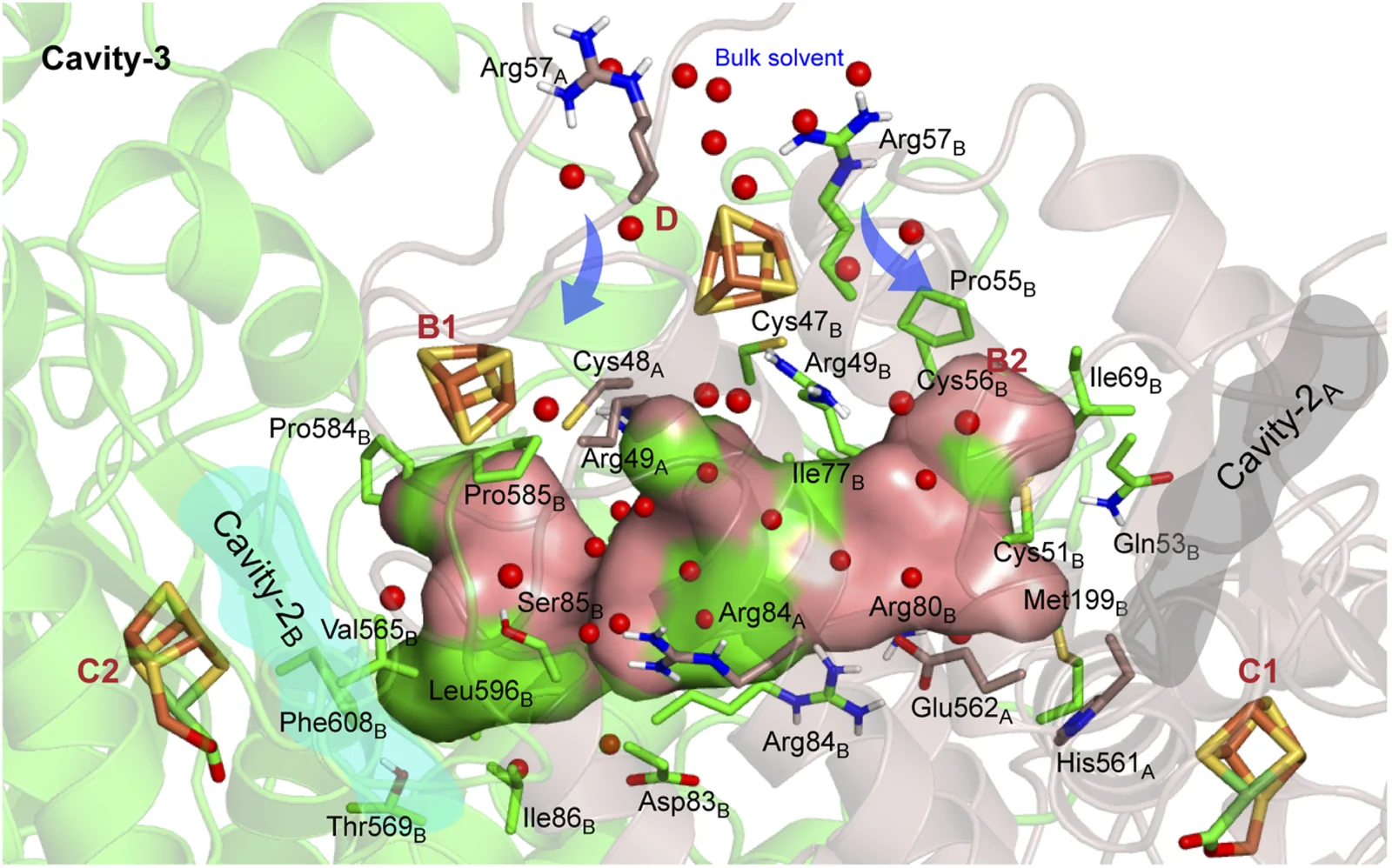
Detailed representation of the amphipathic cavity network associated with Cavity-3, highlighting its structural organization and the origin of water flow pathways leading to and within the pocket. Green patches at the surface showcase the hydrophobic region and light red color shows the hydrophilic region.

Cavity-2_A_ consists primarily of hydrophobic residues from subunit-A, including Ala556_A_, Met560_A_, Val582_A_, Leu583_A_, Pro584_A_, Pro585_A_, Ala564_A_, Val565_A_ and Ile580_A_. Within this highly hydrophobic environment during MD, very few traces of water molecules were observed, likely regulated by the acidic residue Glu43_A_, which contributes to the localized polarity required for water stabilization. Similarly, Cavity-2_B_ is composed predominantly of hydrophobic residues from subunit-B, including Ile86_B_, Leu209_B_, Leu210_B_, Cys215_B_, Thr669_B_, Val565_B_, Ala564_B_, Ala566_B_, Ile567_B_, Ala571_B_, Leu596_B_, Val600_B_, and Phe608_B_ (Fig. 9). Despite its high hydrophobicity, two water molecules were observed within the pocket. These water molecules appear to be stabilized by polar residues Ser216_B_, Thr569_B_, and Thr573_B_, which provide localized hydrophilic interactions. Notably, residue Phe608_B_ is the connecting residue between the B1-cluster to C2-cluster through the cavity, as observed in proximity to Cavity-3 (Fig. 10). This suggests a functional continuity between these pockets in facilitating substrate and solvent transport. The functional roles of these two pockets, as indicated by their positioning and composition, are likely centered on providing a pathway for hydrophobic substrate molecules to access the catalytic clusters while enabling minimal yet crucial solvent flow.

#### 3.2.3. Dynamically characterized amphipathic Cavity-3

Among the identified pockets in CODH, Cavity-3 stands out as the largest (volume of 1160.49 Å^3^) and highly persistent cavity within the enzyme. This pocket is structurally unique and functionally significant, as it directly connects all the relevant metal clusters, specifically the B-cluster and D-cluster, essential for electron transfer and catalysis. To our knowledge, this is the first study to bring this cavity into focus, though faint traces of its existence can be observed in the crystal structure,^8^ albeit it was not previously discussed. The nature of this cavity may hint at a possibly crucial functional role, particularly in a system like CODH that implicates both hydrophobic (CO_2_/CO) and hydrophilic (H_2_O) molecules in catalysis.

Cavity-3 exhibits dual characteristics, with a mixed amphipathic surface: a hydrophobic region (green) and a hydrophilic region (red). This amphipathic nature is quantified by its 136 polar alpha spheres out of a total of 191, indicating significant polarity. Its polarity score (Table S5) further corroborates this observation, marking this tunnel as polar-dominant in contrast to the predominantly hydrophobic cavities documented thus far close to the metal clusters.^8^ This identified cavity could be suspected for dual behavior, aligning with the enzymatic requirements for catalytic function. Solvent entry associated with Cavity-3 originates from the bulk and enters the enzyme through the protein matrix close to the D-cluster, the fused region between the two subunits. The positively charged residues Arg57_A_ and the corresponding Arg57_B_ in the other subunit play important roles in directing water into the Cavity-3 network. Notably, these residues also contribute to stabilization of the D-cluster through inter-subunit interactions with Cys39_A_ and Cys39_B_, respectively, after which the water pathway emerging from the D-cluster bifurcates into two distinct branches leading towards the B1 and B2 clusters (Fig. 10). This bifurcation highlights the cavity's ability to efficiently hydrate multiple metallocofactor sites.

The water flow through this cavity is regulated by an array of charged and polar residues, including Cys48_A_, Arg49_A_, Arg84_A_, His561_A_, Glu562_A_, Cys47_B_, Arg49_B_, Cys56_B_, Arg80_B_, Asp83_B_, Arg84_B_, and Ser85_B_, which create a highly coordinated hydrophilic environment. Simultaneously, the hydrophobic surface is maintained by residues of subunit-B such as Ile77_B_, Pro584_B_, Pro585_B_, Leu596_B_ and Phe608_B_, with contributions from the aliphatic side chains of Arg84_A_ and Arg84_B_.

This intricate combination of polar and hydrophobic features underscores the functional versatility of Cavity-3. Importantly, it provides a direct connection between the major electron transfer metal clusters (B1, B2, and D-cluster) *via* organization of a water network. This finding aligns with observations in other enzymatic systems, where water flow between electron transfer cofactors enhances the efficiency of electron transport.^68–70^

Overall, the cavity analysis reveals an evolutionary optimized architecture in which the clusters across the two subunits are interconnected by predominantly hydrophobic cavities, while in parallel the enzyme maintains dedicated water pathways, highlighting a coordinated amphipathic design that supports both substrate movement and water delivery in spatial proximity.

### 3.3. Hydration analysis

**Fig. 11:**
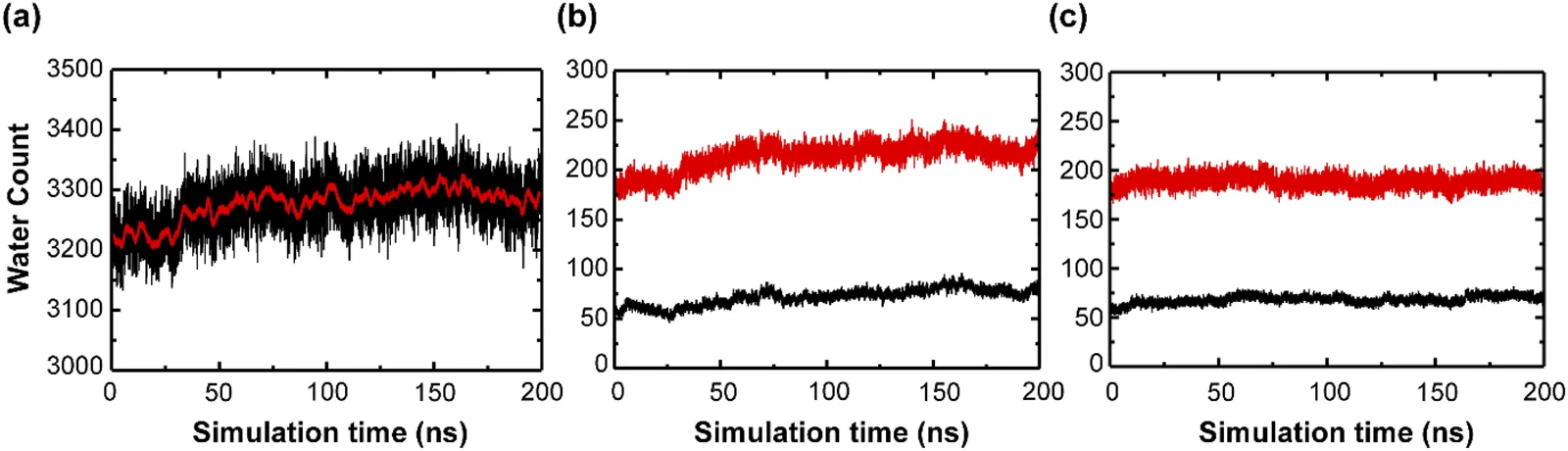
Time evolution of water occupancy during the MD simulation. (a) Total water content within the 3.5 Å region from the protein surface. (b) Water occupancy within 15 Å (black lines) and 20 Å (red lines) of the C1 cluster. (c) Water occupancy within 15 Å (black lines) and 20 Å (red lines) of the C2 cluster.

As evident from the preceding discussion, water channels and hydrophobic cavities are intertwined within CODH, reflecting the requirements of the reaction CO + H_2_O ⇄ CO_2_ + 2H^+^ + 2e^−^ that is catalyzed by the enzyme. For the CO/CO_2_ interconversion, water access to the C-cluster and proton transfer are likely related, but they should not be assumed to occur through the same cavity or pathway.^5,71,72^ The demand of local hydration around the C-cluster is met by the enzyme's structure, which demonstrates significant hydration as revealed by our MD results (Fig. 11a). We further investigated the spatial distribution of water molecules surrounding the enzyme as a whole and around the critical metallocofactors.

The diffusion of bulk water into internal cavities occurs on prolonged timescales, necessitating extensive sampling of protein conformational dynamics.^19,73–75^ Therefore, to comprehensively explore both the dynamics of internal water and the diffusion of bulk solvent, we analyzed the hydration state of CODH based on our 200 ns MD trajectory. By quantifying hydration within a 3.5 Å radius from the protein surface, we observe that the water count around CODH is almost double (∼3300 water molecules, Fig. 11a) compared to the crystallographic water content (∼1591 water molecules). In Fig. 11a, the black curve represents the instantaneous water count during the MD trajectory, while the red curve represents the running/time-averaged water count. Thus, the time-averaged count of ∼3300 water molecules observed during the MD simulation reflects the dynamically hydrated state of the same region of 3.5 Å radius from the protein surface. Within a 15 Å radius of each C-cluster (black lines in Fig. 11b and c), an average of ∼71.7 (for the C1-cluster) and ∼68.1 (for the C2-cluster) water molecules were observed, substantially higher than the 46 water molecules identified in the crystallographic model. The red line represents the water count obtained using an expanded region up to 20 Å, which includes the additional bulk-solvated water molecules around the cluster. This enhanced hydration, both within the internal cavities and on the protein surface, underscores the need for a detailed investigation into the pathways through which bulk water penetrates the protein and reaches the C-cluster.

### 3.4. Water dynamics around the C-cluster

**Fig. 12:**
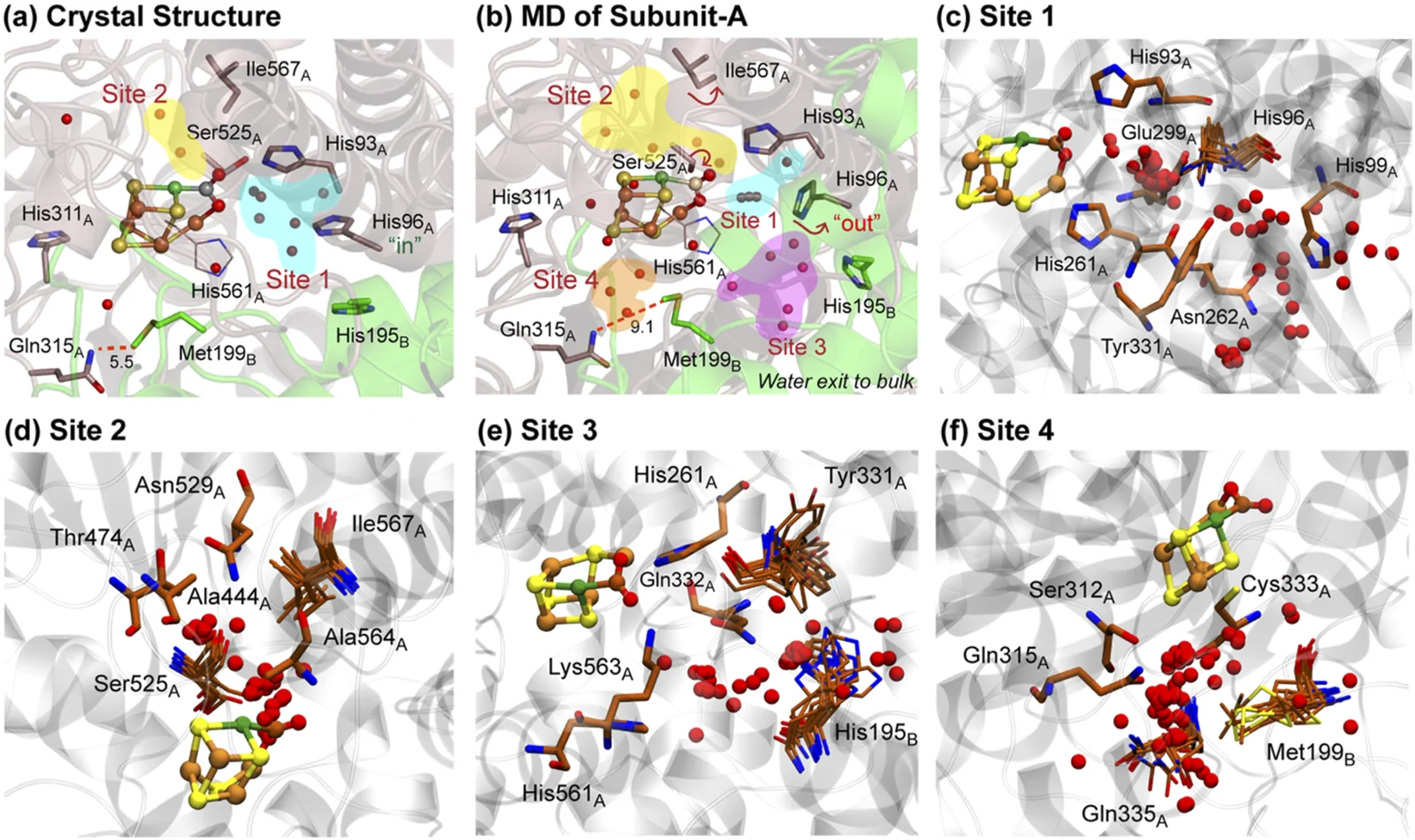
(a) Crystal-structure water molecules showing the core hydration sites 1 and 2. (b) MD-derived hydration pattern of subunit-A, showing retention of sites 1 and 2 together with two additional dynamic sites, 3 and 4. (c–f) Time averaged water positioning for sites 1–4 over successive 20 ns intervals. The C-cluster is shown as a ball and stick model, and red spheres indicate water oxygen positions; selected nearby residues are shown in sticks.

The crystal structure (Fig. 12a) reveals two hydration sites in the vicinity of the C-cluster. Site 1 is located near the substrate-binding region, in front of the CO_2_ binding site, and comprises six water molecules. Site 2 is proximal to the Ni center and is defined by two closely positioned water molecules. Comparison with the crystal structure shows that the core hydration pattern near the C-cluster is largely retained in our MD ensemble (Fig. 12b), while additional localized hydration features become apparent during the simulation. In the vicinity of the C-cluster, site 1 remains positioned near the CO_2_ binding region and site 2 near the Ni-proximal side, indicating that these two regions represent persistent elements of the local hydration framework. In addition, two further hydration features, denoted as site 3 and site 4, emerge from the MD data. Because the cumulative water distribution in Fig. 12b is spatially broad, average water positions were further examined in successive 20 ns windows over the 200 ns trajectory. This time-resolved view indicates that the observed solvent organization is better described as a set of recurrent localized water clusters or hydration pockets, rather than as fully continuous water channels. A corresponding analysis was also performed for subunit-B and revealed a comparable overall pattern, including both persistent core hydration sites and additional dynamically emerging hydration sites (Fig. S5). On this basis, the four sites were analyzed individually, with attention to their structural location and to the nearby residues that shape local water accessibility.

Site 1 is the most clearly retained hydration feature and appears to correspond to the best-defined water cluster already visible in the crystal structure. Crystallographically, this region contains a water cluster adjacent to the CO_2_ binding side of the C-cluster and near His93_A_ and His96_A_. In the MD ensemble, this hydration feature remains present and extends into a broader local aqueous region lined by His93_A_, His96_A_, His99_A_, Glu299_A_, His261_A_, Asn262_A_, and Tyr331_A_ (Fig. 12c). The extension of this cluster coincides with reorientation of the imidazole side chain of His96_A_ from the inward to the outward conformation (Fig. S6), which opens additional local space toward the protein surface. In this configuration, Asn262_A_, Tyr331_A_, and His99_A_ define the outer part of the hydration feature where it approaches bulk solvent. At present, this result supports site 1 as a persistent and surface-connected hydration region near the CO_2_ binding side of the active site. While this makes it a plausible region for local solvent access, the present analysis does not establish a unique transport pathway or a specific functional role beyond its recurrent structural presence.

Site 2 is also retained relative to the crystal structure, but it remains more compact and spatially restricted than site 1. In the simulation, the cluster near this region stays close to the Ni-proximal side of the C-cluster and is shaped mainly by Ser525_A_, Ile567_A_, Ala564_A_, Thr474_A_, and Asn529_A_ (Fig. 12d). Small extensions of the local water cluster are observed in association with conformational adjustments of Ile567_A_ and Ser525_A_, (Fig. S7 and S8) but the feature remains confined and does not show a clear connection to the protein surface. In structural terms, this limited extent likely reflects local steric closure by surrounding side chains, particularly around the Asn529_A_ and Thr474_A_ regions, which restricts further propagation of the water cluster. Thus, site 2 appears to represent a localized Ni-proximal hydration pocket rather than an extended solvent-connected feature.

In contrast to sites 1 and 2, site 3 is not evident in the crystal structure but emerges reproducibly in the MD ensemble as a well-defined and distinct hydration feature (Fig. 12e). This region is supported by residues His195_B_, Tyr331_A_, His261_A_, Lys563_A_, His561_A_, and Gln332_A_ and lies adjacent to the broader aqueous region associated with site 1. The shared involvement of Tyr331_A_ suggests that site 3 is not fully independent of site 1, but rather forms part of a neighboring hydration region sampled only in the dynamic ensemble. Even so, the water molecules occupying site 3 remain locally clustered and do not approach the CO_2_ binding region directly, as access in that direction appears restricted by Gln332_A_ and Lys563_A_. Site 3 is therefore best interpreted as a dynamically formed local hydration pocket that becomes accessible through protein motion, rather than as a crystallographically preformed cavity or a demonstrated transport route.

Site 4 represents an additional localized hydration feature observed in the MD ensemble near the Ser312_A_, Gln315_A_, Cys333_A_, and Gln335_A_ regions and toward Met199_B_ (Fig. 12f). As with site 3, this feature is not resolved as a defined water cluster in the crystal structure but appears recurrently in the simulation. The position of this site suggests that it belongs to the broader dynamic hydration landscape surrounding the C-cluster rather than to the core crystallographic water network itself. Taken together, the four sites indicate that the C-cluster environment is organized around a conserved core hydration pattern, represented by sites 1 and 2, together with additional dynamic hydration pockets, represented by sites 3 and 4, that become populated through local side-chain fluctuations. At present, these results support the existence of recurrent local hydration features near the active site of CODH.

### 3.5. Interplay between histidine protonation and metal cluster solvation

The protonation state of titratable residues such as His, Asp and Glu plays a key role in shaping hydration pathways within the enzyme.^76–78^ In CODH, His93 is an important second-sphere residue because it is located very close to the substrate-binding site and can influence substrate stabilization through its interactions.^9,31,64,79^ In agreement with this, our cavity analysis, water-dynamics analysis around the cluster, and previous experimental^7,9^ and theoretical^29,31,64^ studies together show that His93 plays a central role in shaping the local active-site environment, as discussed in the previous section.

**Scheme 1:**
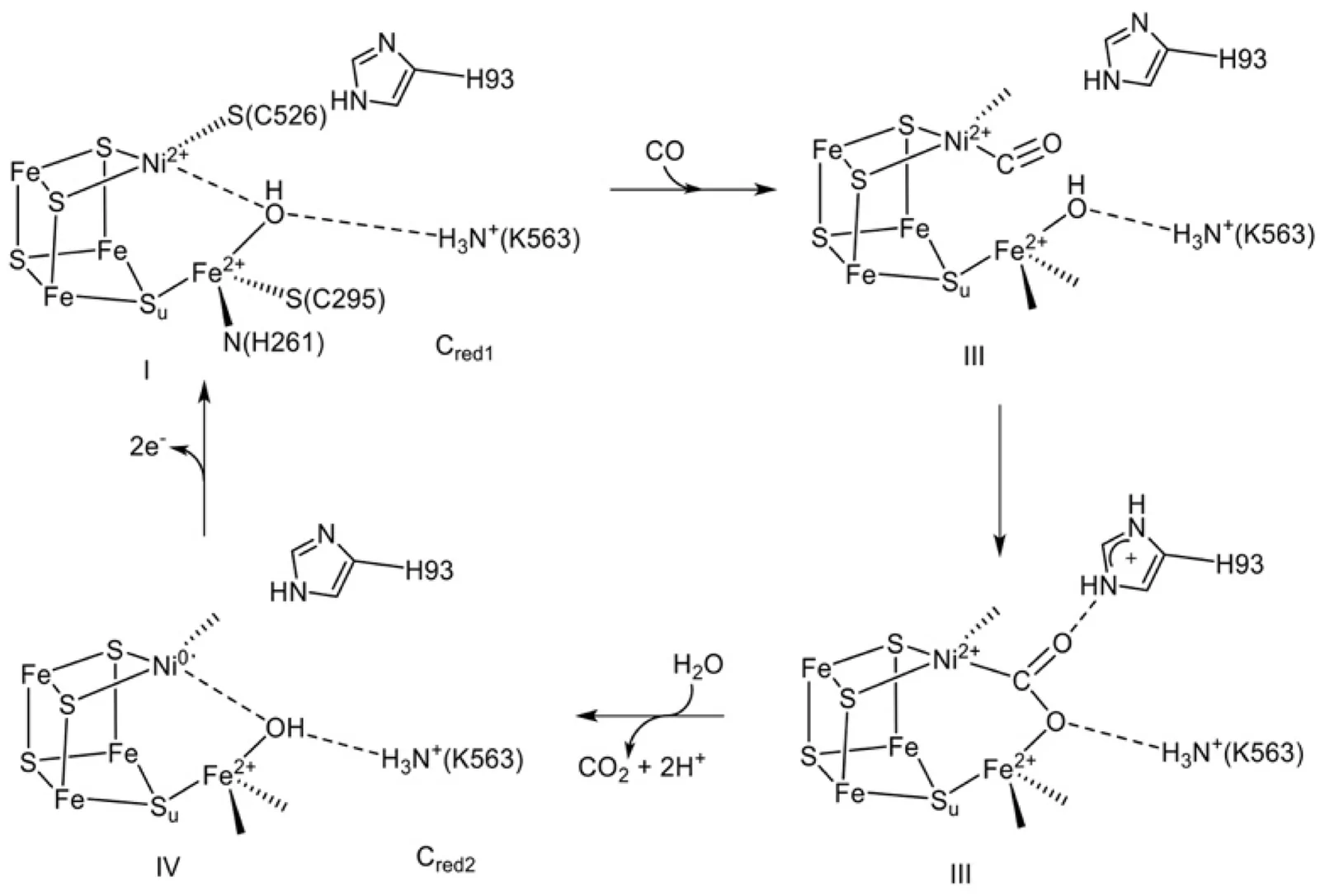
Proposed mechanism^7,31,64^ of CO to CO_2_ interconversion involving His93 as a proton source.

The computationally predicted p*K*_a_ (with the PROPKA^42^ software) of His93 (12.56) significantly exceeds the crystallographic pH (8.0),^9^ suggesting a predominantly protonated state, consistent with mutagenesis studies^7^ as well as past computational (MD,^29^ QM/MM-MD,^31^ and QM/MM^64^) studies. In the reported studies, the protonated state of His93 complemented its proposed role in proton transfer^31^ and its positioning along the putative substrate/water access pathway.^29^ Another crystallographic study suggested that His93 plays a dual mechanistic role during catalysis at the C-cluster (Scheme 1).^9,31^ In the catalytic mechanism of CODH (Scheme 1), for the “C_red1_” state, His93 appears to help gate CO entry by controlling access to the substrate-binding region near the Ni center.^9^ Once the substrate binds at the cluster site, His93 reorients toward the bound intermediate and provides second-sphere stabilization through hydrogen-bonding interactions, which likely helps position and polarize the substrate for subsequent transformation. Despite the support for a protonated His93, alternative protonation states must be considered because the local electrostatic environment of an active-site histidine may be significantly altered through reorganization of hydrogen-bond networks and water repositioning during catalysis. Therefore, to probe the effect of His93 protonation, we simulated three protonation states: HID (δ-protonated), HIE (ε-protonated), and HIP (doubly protonated).

We analysed the distribution of water molecules within 3.5 Å and 5.0 Å (first and second solvation shells, respectively) from the imidazole ring of His93 and the NH_3_^+^ group of Lys563 (Table 1). In the HIP state, His93 formed a stable hydrogen bond with the oxygen of C-cluster-bound CO_2_*via* its ε-proton, while preserving the two key structured hydration sites discussed in the previous section: site 1, oriented toward His93, and site 2, located proximal to the Ni center. This well-organized hydration environment supports persistent water flow and proton relay, with average water occupancies of 2.29 (first shell) and 5.71 (second shell).

**Table 1:** Average water occupancy (first and second solvation shells) and RMSF values (in Å) of His93 and Lys563 for the three simulated protonation states (HID, HIE, and HIP) of His93. The values are reported separately for each subunit

	Model HIP	Model HIE	Model HID
RMSF (His93)	0.39_A_/0.39_B_	0.51_A_/0.80_B_	0.67_A_/0.79_B_
RMSF (Lys560)	0.44_A_/0.43_B_	0.49_A_/0.47_B_	0.52_A_/0.46_B_
Avg. water (around 3.5 Å)	2.29_A_/2.61_B_	5.46_A_/5.58_B_	9.39_A_/8.46_B_
Avg. water (around 5.0 Å)	5.71_A_/5.68_B_	9.17_A_/10.28_B_	13.64_A_/13.56_B_

**Fig. 13:**
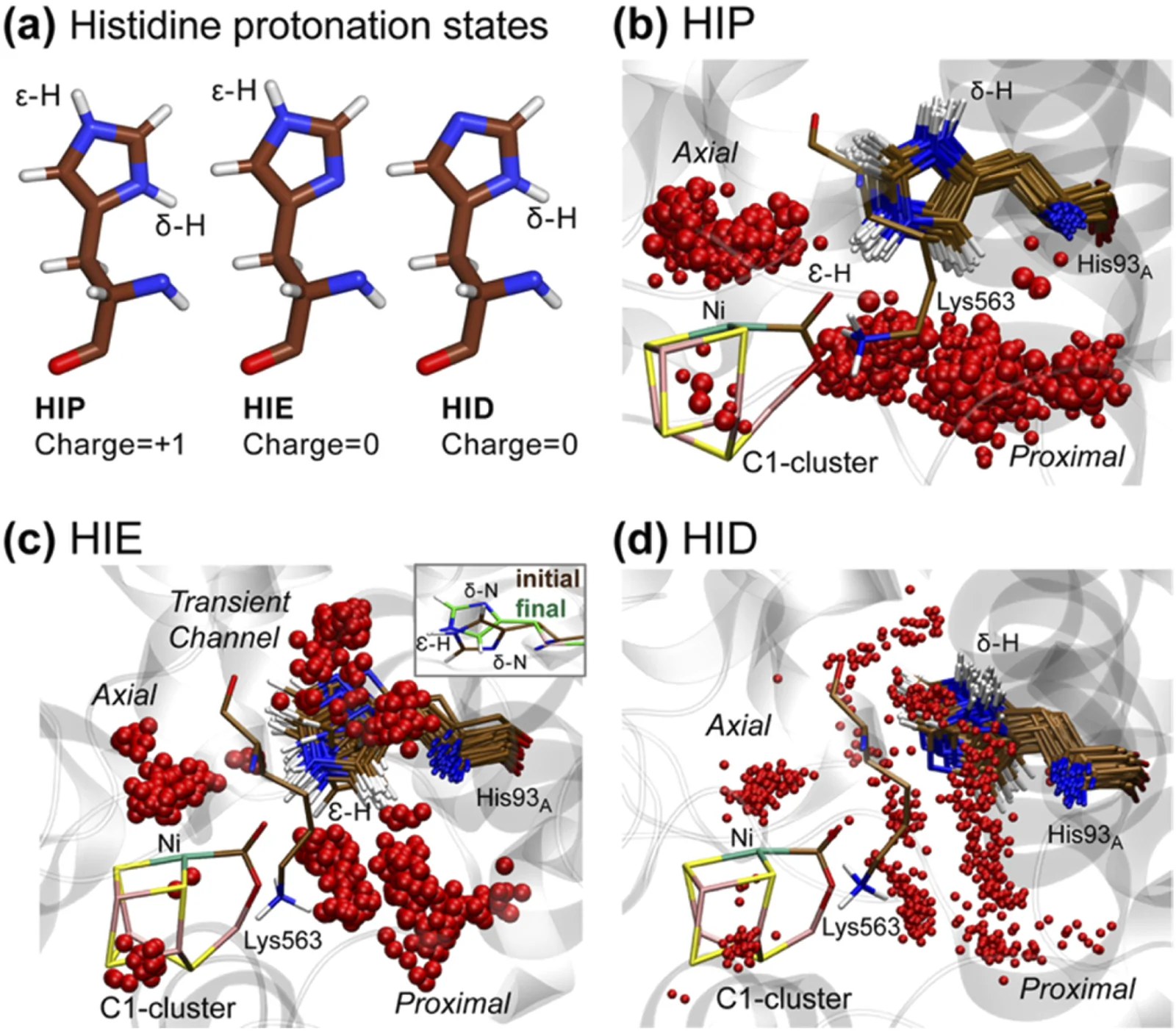
(a) Possible protonation states of histidine: HIP, HIE and HID. Superposition of MD frames illustrating water molecule positions within 5 Å of the imidazole ring of His93 and the NH_3_^+^ group of Lys563 across the three protonation states: (b) doubly protonated His93 (HIP), (c) ε-protonated His93 (HIE), and (d) δ-protonated His93 (HID). Red spheres represent water molecules, and the C-cluster is shown for spatial reference. Axial and proximal directions are marked to emphasize the preferential water positions. Note that due to the conformational change in the imidazole ring of His93 in the HIE state, additional transient water locations were mapped. The distance between His and CO_2_ is reported in Fig. S9.

The HIE state exhibited comparable behaviour, maintaining hydrogen bonding with CO_2_, but also revealed conformational heterogeneity. Two distinct rotamers of His93 were observed. In the initial conformation, no hydration at the δ-N site was detected due to proximal water crowding. Over time, water accumulation and local flexibility (RMSF = 0.51 Å) induced a flip of the imidazole ring, allowing transient water molecules to occupy the δ-N vicinity. This conformational shift resulted in average water occupancies of 5.46 and 9.17 in the first and second solvation shells of subunit A (Fig. 13).

In contrast, the HID form, lacking the ε-proton, was unable to sustain hydrogen bonding with CO_2_ and exhibited the highest structural flexibility (RMSF = 0.67 Å). The absence of this interaction opened the proximal region, allowing continuous water penetration and leading to elevated hydration levels (9.39 and 13.64 water molecules in the respective solvation shells) yet with reduced structural organization. Throughout all simulations, Lys563 maintained a stable interaction with the CO_2_ oxygen, exhibiting minimal fluctuation (0.43–0.52 Å), reinforcing its H-bond strengthening role near the C-cluster.

Collectively, these findings highlight the critical role of His93 protonation in establishing and maintaining an efficient hydration network conducive to proton transfer. Among the states examined, the HIP conformation provides the most catalytically favourable environment by preserving hydrogen bonding with CO_2_ (Scheme 1), maintaining defined hydration sites, and optimizing the donor–acceptor geometry required for effective proton delivery.

## 4. Conclusions

In this study, we conducted extensive large-scale molecular dynamics simulations of fully hydrated carbon monoxide dehydrogenase (CODH) to better understand structurally and functionally important internal features. The investigation focused on four key aspects: (i) structural connections of protein subunit matrices near the metal clusters, (ii) mapping key cavities in the proximity of metal clusters, (iii) hydration of the enzyme, particularly around the catalytic C-cluster, and (iv) the effect of the protonation state of the functionally crucial residue His93 on active site solvation. Upon fusion of the two monomeric subunits, we observed significant mutual interactions near the metal clusters, particularly the D-cluster, supported by an enhanced hydrogen bonding network between residues from the two subunits, contributing to the enzyme's overall stability.

Analysis of MD simulations provided detailed mapping of cavities and water channels around the metal clusters, uncovering a parallel and entangled system of hydrophobic and hydrophilic routes that suggest the efficient delivery of substrates (CO_2_/CO) and water to the catalytic C-cluster. The amphipathic environment formed by hydrophobic and polar residues likely demonstrates the evolutionary optimization of the enzyme in balancing the distinct requirements of substrate binding and water transport. Additionally, surface-exposed residues Arg57_A_ and Arg57_B_ facilitate the bifurcation of bulk-solvent from the D-cluster towards B1 and B2-cluster sites. Through inter-subunit interactions of Arg57_A_–Cys39_B_ and Arg57_B_–Cys39_A_, these residues stabilize the D-cluster and bridge the D-cluster with the B1/B2-cluster (involved in electron-transfer) through solvent flow.

The simulations displayed nearly twice the level of hydration compared with that inferred from crystallographically resolved water molecules, underscoring the ability of the enzyme to sustain a dynamic yet functionally important hydration network. Quantitative mapping revealed increased hydration near the C-clusters. The dynamic conformational changes of catalytically crucial residues such as His96, Ile567, and Ser525 reveal new hydration sites in the vicinity of the C-cluster that enable identification of specific water transfer pathways. Finally, the protonation state of His93 was shown to critically determine the organization of water networks near the C-cluster, impacting the formation of plausible water channels essential for proton transfer. However, His93 protonation and dynamics may differ for other catalytic states, such as hydroxo/water-bound or CO-bound intermediates, depending on the redox state (C_red1_/C_red2_) and ligand identity of the C-cluster.

The observed interplay between dynamically regulated hydration sites and residue protonation equilibria establishes a framework for future mechanistic studies of CODH and its catalytic intermediates.^9,64^ Our comprehensive molecular-level analysis reveals that entangled hydrophobic and hydrophilic channels cooperatively govern catalytic readiness, a concept warranting further exploration from both evolutionary and mechanistic perspectives. These insights can inform the analysis of analogous enzymatic/bioinorganic systems and also inspire future efforts in protein engineering and rational catalyst design for carbon capture, particularly in the context of functional matrix optimization. Overall, this work deepens our molecular understanding of enzymatic CO_2_ processing and holds broader relevance for global carbon cycling and the development of sustainable carbon management strategies.

## Author contributions

S.·Y.: methodology, investigation, analysis, writing – original draft; D. A. P.: conceptualization, methodology, supervision, writing – review and editing.

## Conflicts of interest

There are no conflicts to declare.

## Supplementary Material

SC-OLF-D6SC04231G-s001

## Data Availability

Original data from this work are provided as an open-access data set hosted by Edmond, the Open Research Data Repository of the Max Planck Society at https://doi.org/10.17617/3.AUOKHW. Supplementary information (SI): Fig. S1–S9 and Tables S1–S5. See DOI: https://doi.org/10.1039/d6sc04231g.
