# Supplementary material for: Entangled hydrophobic and hydrophilic networks in carbon monoxide dehydrogenase
Source: Chem Sci. 2026 Aug 30;17(41):19879–94. doi: 10.1039/d6sc04231g (PMC13543214; doi:10.1039/d6sc04231g)
Supplement: Supplementary file 1 [file SC-017-D6SC04231G-s001.pdf]

Supplementary Information (SI) for Chemical Science.
This journal is © The Royal Society of Chemistry 2026

Electronic Supplementary Information

Entangled Hydrophobic and Hydrophilic Networks in Carbon Monoxide Dehydrogenase

Shalini Yadav, Dimitrios A. Pantazis**

Max-Planck-Institut für Kohlenforschung, Kaiser-Wilhelm-Platz 1, 45470 Mülheim an der Ruhr, Germany
* Email: yadav@kofo.mpg.de; dimitrios.pantazis@kofo.mpg.de

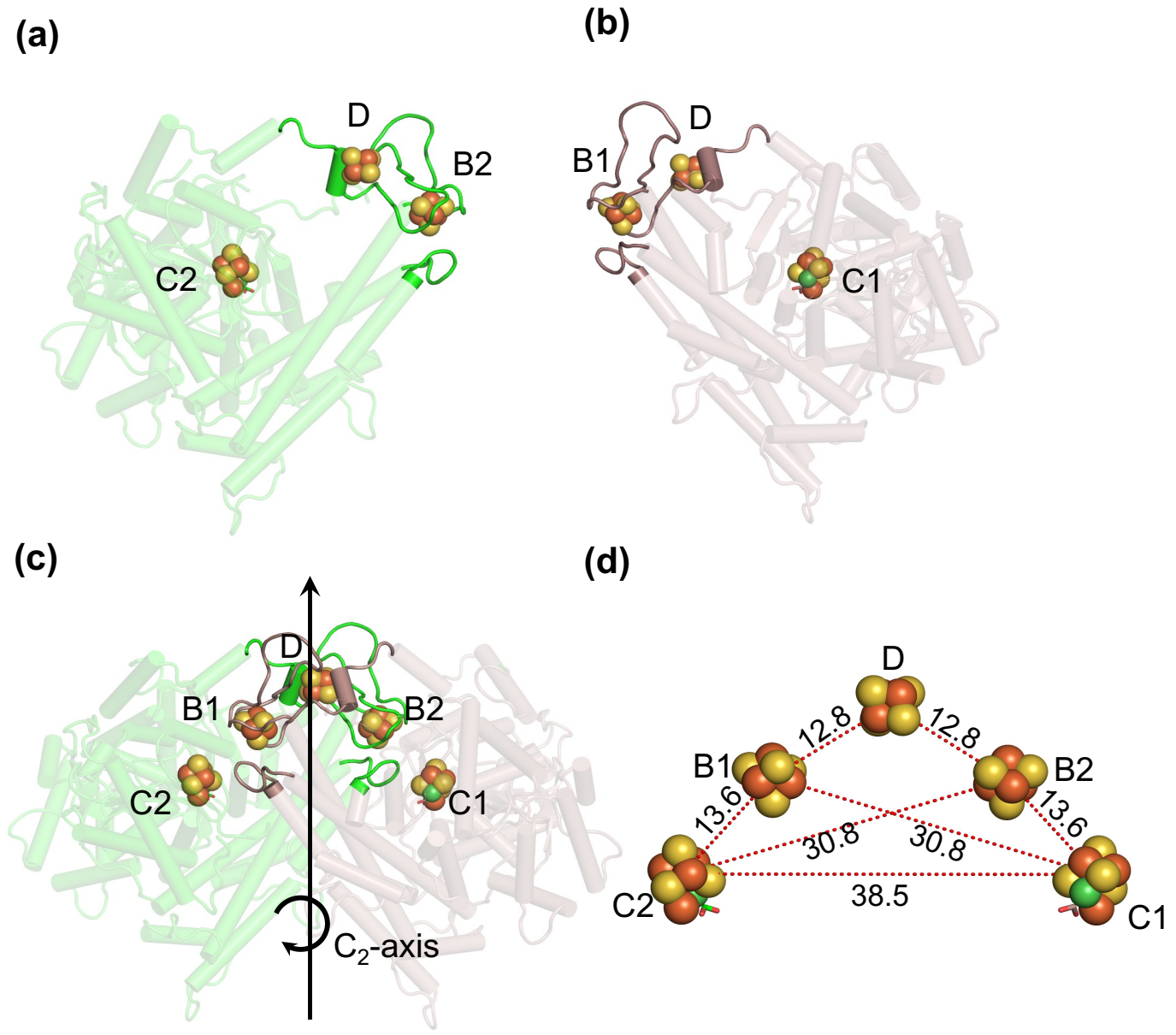

Figure S1. Visual representation of CODH enzymatic assembly: (a) Subunit-B (b) Subunit-A. (c) Biologically assembled unit, (d) Distances between clusters in the dimeric unit.

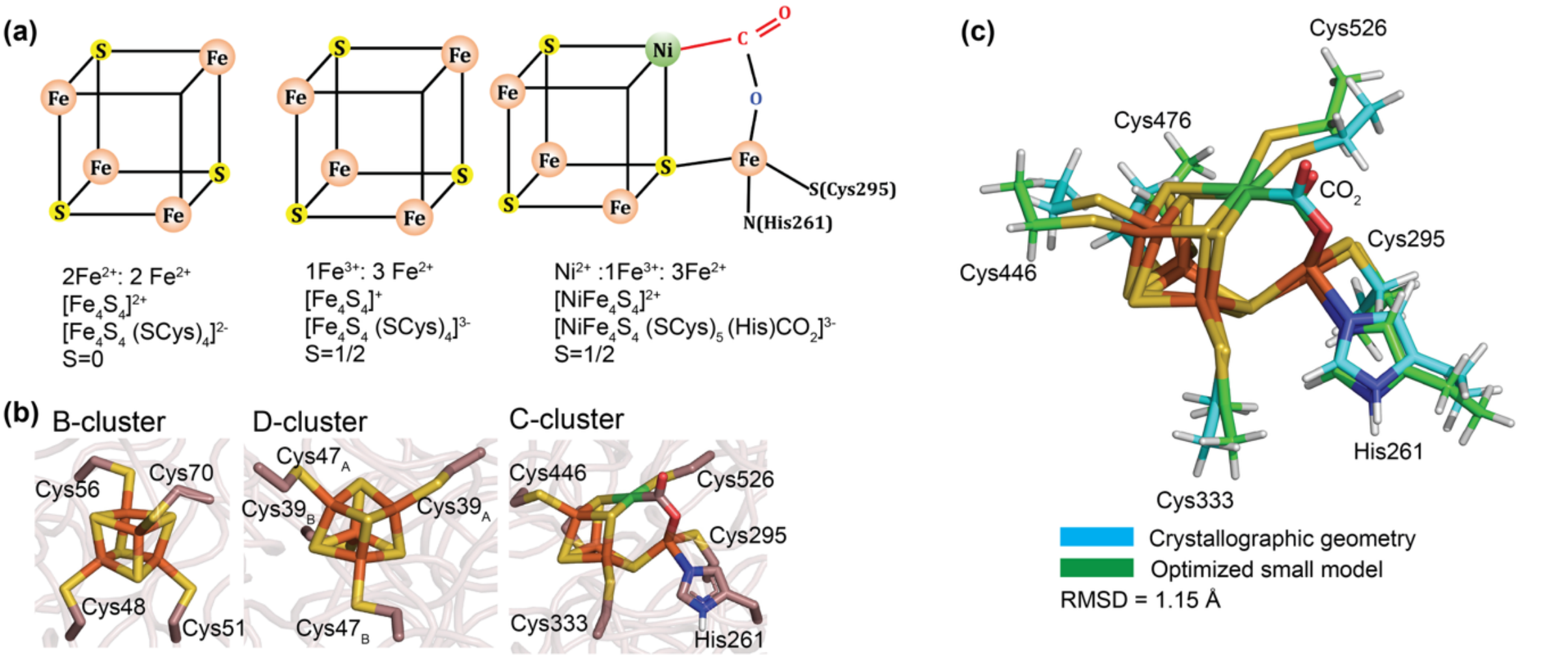

Figure S2. (a) Outlining the structural and electronic characteristics of the B–cluster, D–cluster, and C–cluster utilized in molecular dynamics simulations. (b) 3-D representation of metal cofactor where all Fe and Ni centers of the cofactors are covalently bonded to sulfur atoms of cysteine residues within the protein matrix. (c) Overlay of the crystallographic and optimized small-model geometries for C-cluster. Carbon atoms in the crystallographic structure are shown in cyan, whereas those in the optimized small model are shown in green.

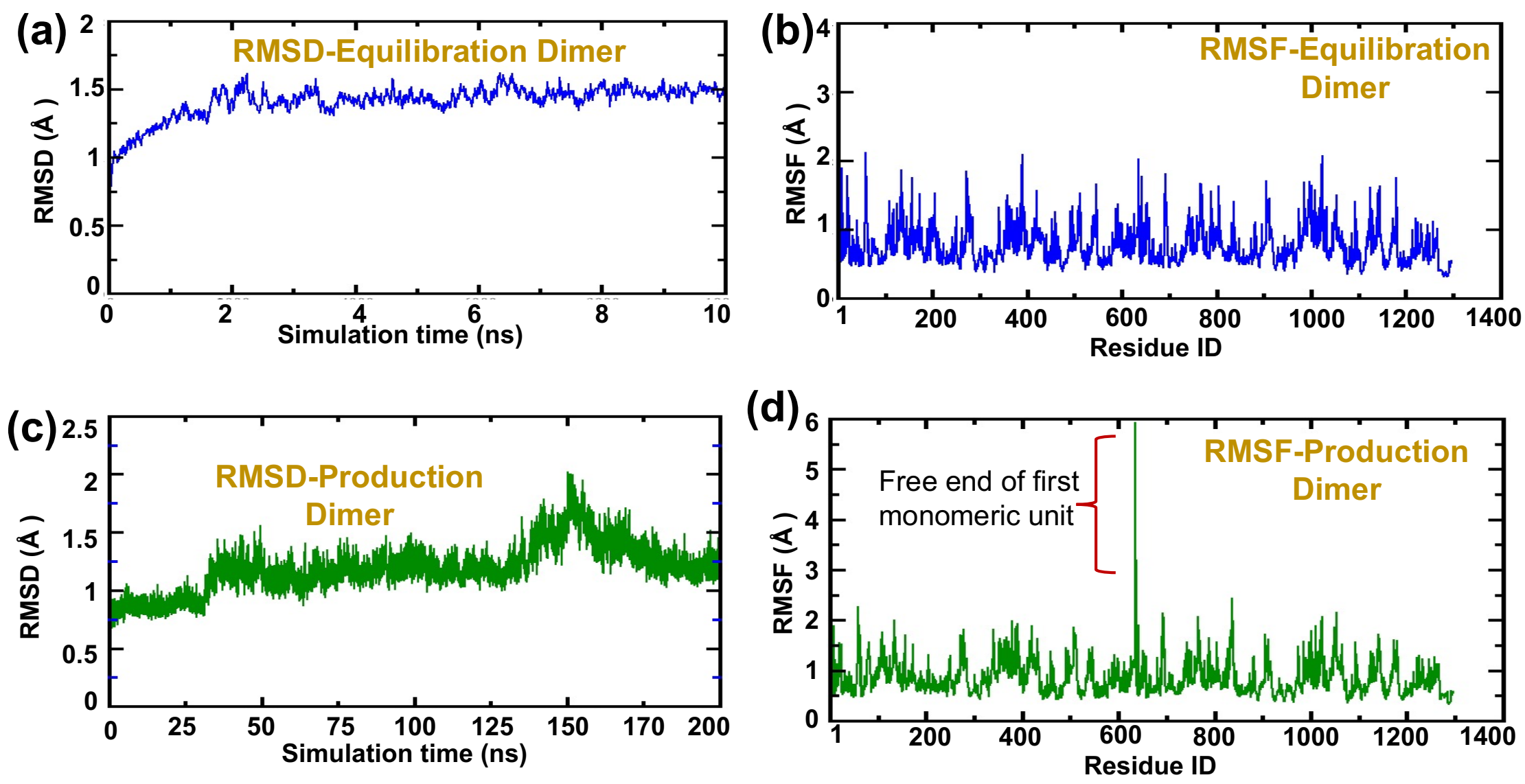

Figure S3. Time evolution of the structural dynamics of the CODH$_{CH}$ complex during equilibration and production MD simulations. The RMSD and RMSF profiles are shown for the 10 ns equilibration phase in panels (a) and (b), respectively, and for the 200 ns production simulation in panels (c) and (d). The enhanced fluctuation around residue ID 634 corresponds to the free terminus of one monomeric unit.

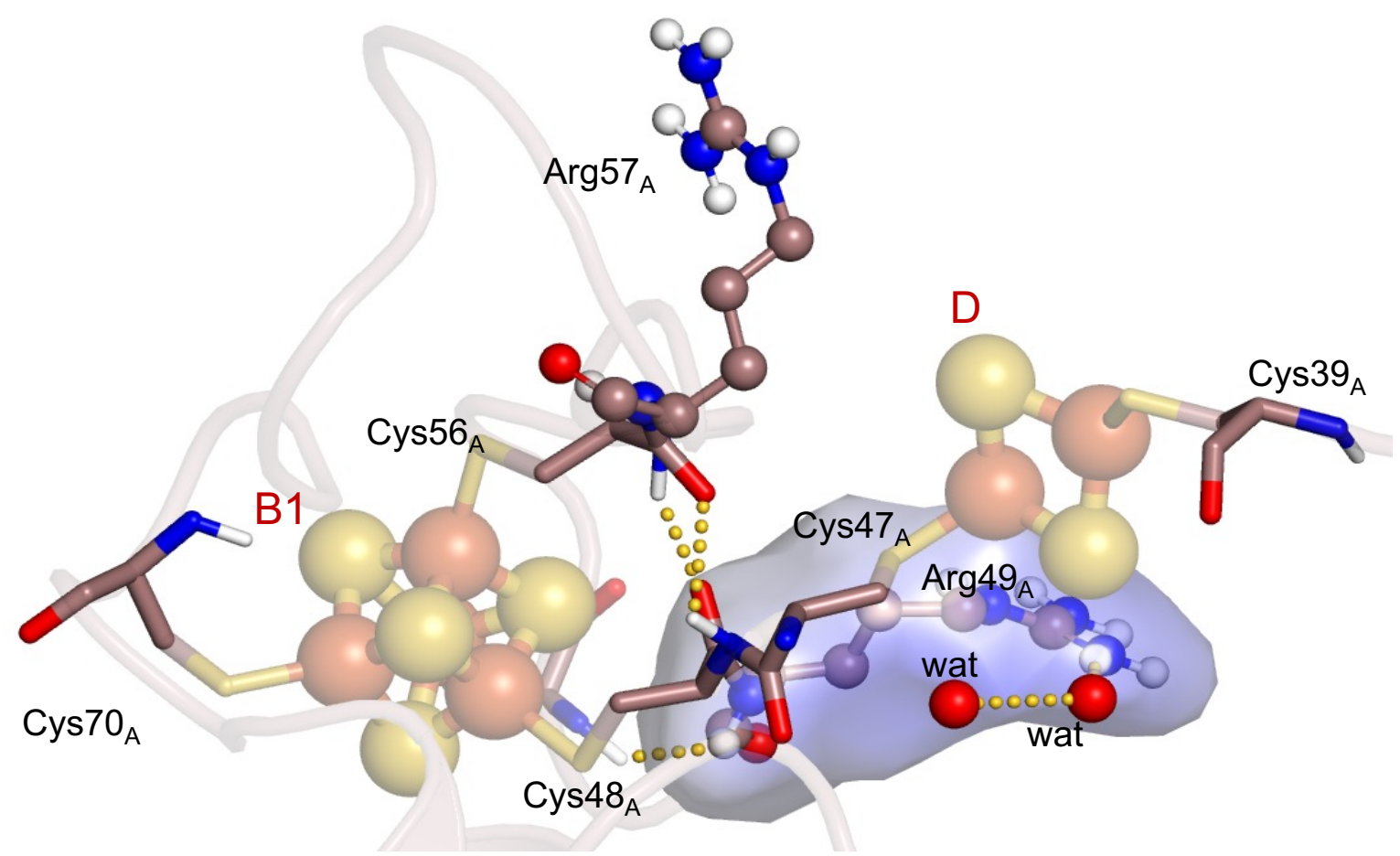

Figure S4. Interactions of residues surrounding the B- and D-cluster within the protein matrix in monomeric unit.

Table S1. Crystallographic data on CODH from different organisms.

	Organism	PDB ID	Resolution (Å)
1	*Rhodospirillum rubrum (Rr)*	1JQK[1]	2.80
2	*Carboxydothermus hydrogenoformans* ($CODH_{CH}$ II)	1SU6[2]	1.64
3	*Carboxydothermus hydrogenoformans* ($CODH_{CH}$ II)	3B51[3]	1.40
4	*Carboxydothermus hydrogenoformans* ($CODH_{CH}$ II)	3B52[3]	1.50
5	*Carboxydothermus hydrogenoformans* ($CODH_{CH}$ II)	3B53[3]	1.50
6	*Carboxydothermus hydrogenoformans* ($CODH_{CH}$ II)	3I39[4]	1.36
7	*Carboxydothermus hydrogenoformans* ($CODH_{CH}$ II)	2YIV[5]	1.28
8	*Carboxydothermus hydrogenoformans* ($CODH_{CH}$ II)	4UDX[6]	1.03
9	*Carboxydothermus hydrogenoformans* ($CODH_{CH}$ II)	4UDY[6]	1.09
10	*Carboxydothermus hydrogenoformans* ($CODH_{CH}$ II)	5FLE[7]	1.23
11	*Carboxydothermus hydrogenoformans* ($CODH_{CH}$ IV)	6ELQ[8]	2.52
12	*Desulfovibrio vulgaris* ($CODH_{Dv}$)	6B6V[9]	2.50
13	*Desulfovibrio vulgaris* ($CODH_{Dv}$)	6ONS[10]	2.48
14	*Desulfovibrio vulgaris* ($CODH_{Dv}$)	6VX1[11]	2.78
15	*Thermococcus sp.* AM4 (Ni-free CODH)	6T7J[12]	2.43
16	*Clostridium autoethanogenum* (CODH/ACS)	6YU9[13]	1.90
17	*Desulfovibrio vulgaris* ($CODH_{Dv}$)	7TSJ[14]	2.10
18	*Carboxydothermus hydrogenoformans* ($CODH_{CH}$/CooS-V)	7B7Q[15]	1.35
19	*Carboxydothermus hydrogenoformans* ($CODH_{CH}$ II-mutant)	7ZX3[16]	1.68
20	*Carboxydothermus hydrogenoformans* ($CODH_{CH}$ II-mutant)	7ERR[17]	2.26
22	*Carboxydothermus hydrogenoformans* ($CODH_{CH}$ II-mutant)	8X9D[18]	2.11

Table S2. MM/GBSA calculated total residue-decomposition energy (in kcal/mol) of the protein matrix in monomeric and dimeric CODH.

	**Total decomposition Energy**
Monomer	-15705.87
Dimer	-31465.15

Table S3. MM/GBSA calculated pair-wise residue interaction energy (kcal/mol) of the residues near the B and D-cluster region.

**Residue of unit A**	**Residue of Unit B**	**Interaction Energy**	**Std. dev.**
Arg31	Ile69	-6.10	1.03
Cys47	Arg49	-4.87	2.38
Arg49	Cys47	-10.19	2.27
Arg57	Met35	-6.99	1.01
Arg57	Pro37	-3.23	1.09
Lys450	Leu52	-3.37	1.49
Lys450	Gly54	-5.91	0.93
Glu562	Arg84	-9.02	0.99
		Total = -49.69	Total = 11.19

Table S4. MM/GBSA calculated pair-wise residue interaction energy (kcal/mol) of the residues near the C-cluster region and beyond.

**Residue of unit A**	**Residue of unit B**	**Interaction Energy**	**Std. dev.**
Cys70	Gln335	-4.38	0.46
His102	Glu156	-4.04	1.71
Glu156	His102	-5.01	0.84
His188	Lys98	-6.62	2.63
His195	Gln332	-3.99	0.84
Arg196	Met355	-3.51	1.51
Asp202	Ser356	-3.32	0.82
Asp202	Lys357	-4.04	0.75
Asn207	Glu354	-3.27	0.64
		Total = -38.16	Total = 10.20

Text S1. ***Fpocket*** **software details and descriptors**

Alpha spheres form the basis of this approach to identifying potential binding pockets on protein surfaces. These spheres are defined as geometric constructs that contact exactly four protein atoms in three-dimensional space without enclosing any other atoms. The program employs alpha spheres to probe concave surface regions, grouping nearby spheres into clusters to delineate potential binding sites. Each sphere is categorized as polar or apolar, depending on the electronegativity of the atoms it contacts. Key properties of these spheres—such as count, radius, and polarity—within a cluster are instrumental in characterizing the local physicochemical environment and contribute to the calculation of descriptive metrics like pocket volume, hydrophobicity and polarity.

- ***Cavity Volume:*** This descriptor estimates the volume of a binding cavity using a Monte Carlo algorithm, which calculates the total volume occupied by all alpha spheres.
- ***Hydrophobicity score and Polarity Score:*** The hydrophobicity descriptor is based on a residue-based hydrophobicity scale published by Monera et al.[19] For all residues implicated in the binding site the mean hydrophobicity score is calculated and is used as descriptor for the whole pocket. Each residue is evaluated only once. Additionally, this descriptor also measures the hydrophilicity character of a binding pocket. To each residue of the binding pocket a polarity score is assigned. The final polarity score is the mean of all polarity scores of all residues in the binding pocket.
- ***Substrate Binding Score:*** The substrate binding score (also referred to as druggability score) evaluates the likelihood of a pocket to accommodate a small molecule. It is represented by a numerical value ranging from 0 to 1, assigned to each pocket using a logistic function. A low score suggests that small molecules are unlikely to bind to the pocket, whereas a (threshold) score of 0.5 indicates a moderate potential for substrate binding and a score of ~1 signifies a high probability of binding.

Table S5. Identified cavities of CODH and their descriptor values.

Pocket number	Druggability score	Volume	Volume Score	Polarity Score	Hydrophobicity	Charge Score
Cavity-1_A	0.004	455.746	3.938	14	1.562	0
Cavity-1_B	0.982	547.373	4.609	0	88.435	0
Cavity-2_A	0.868	391.747	3.556	4	45.778	0
Cavity-2_B	0.883	345.831	3.632	3	51.842	-1
Cavity-3	0.967	1160.49	3.757	13	20.784	7

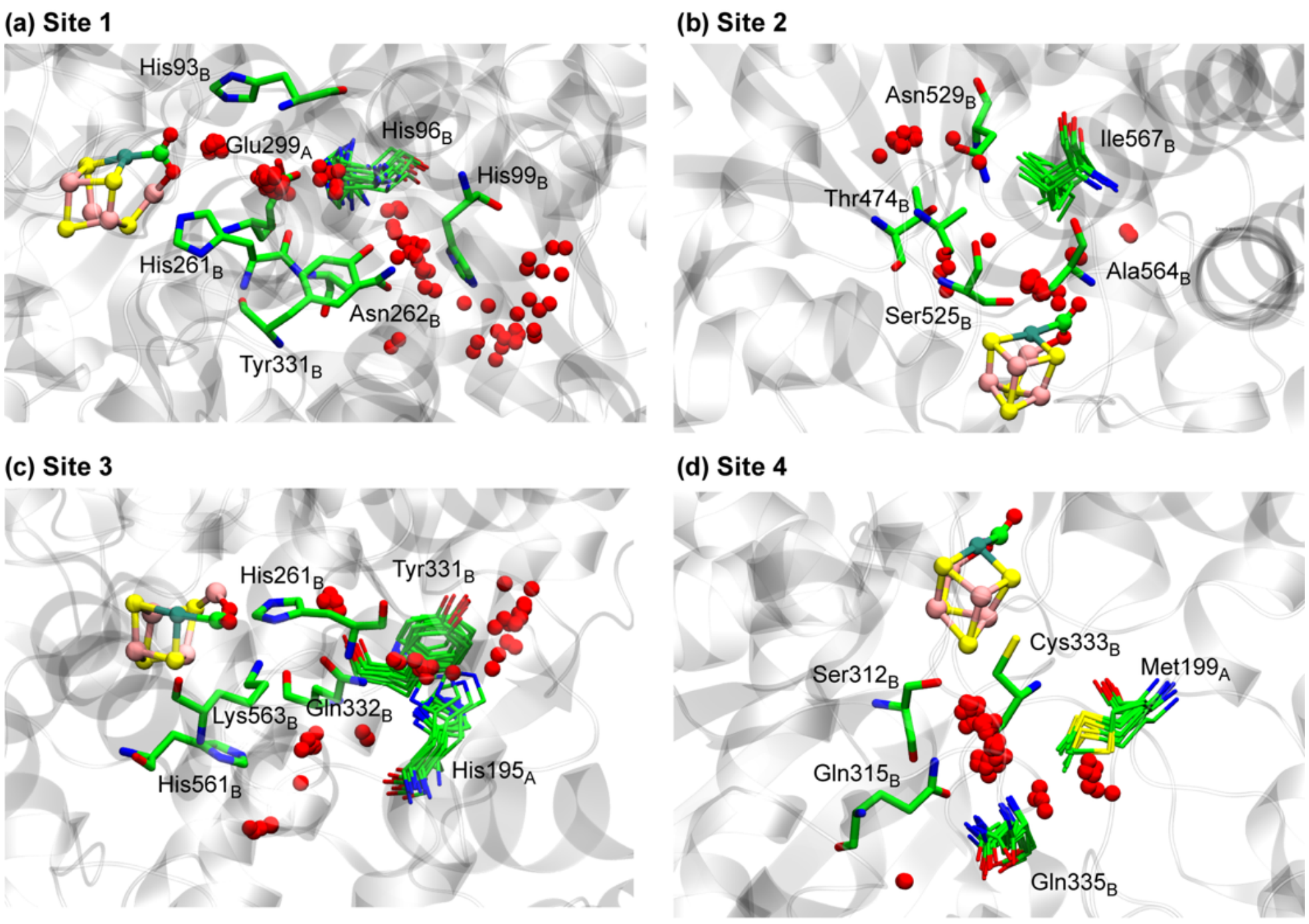

Figure S5. Hydration sites of the C2-cluster in Subunit-B.

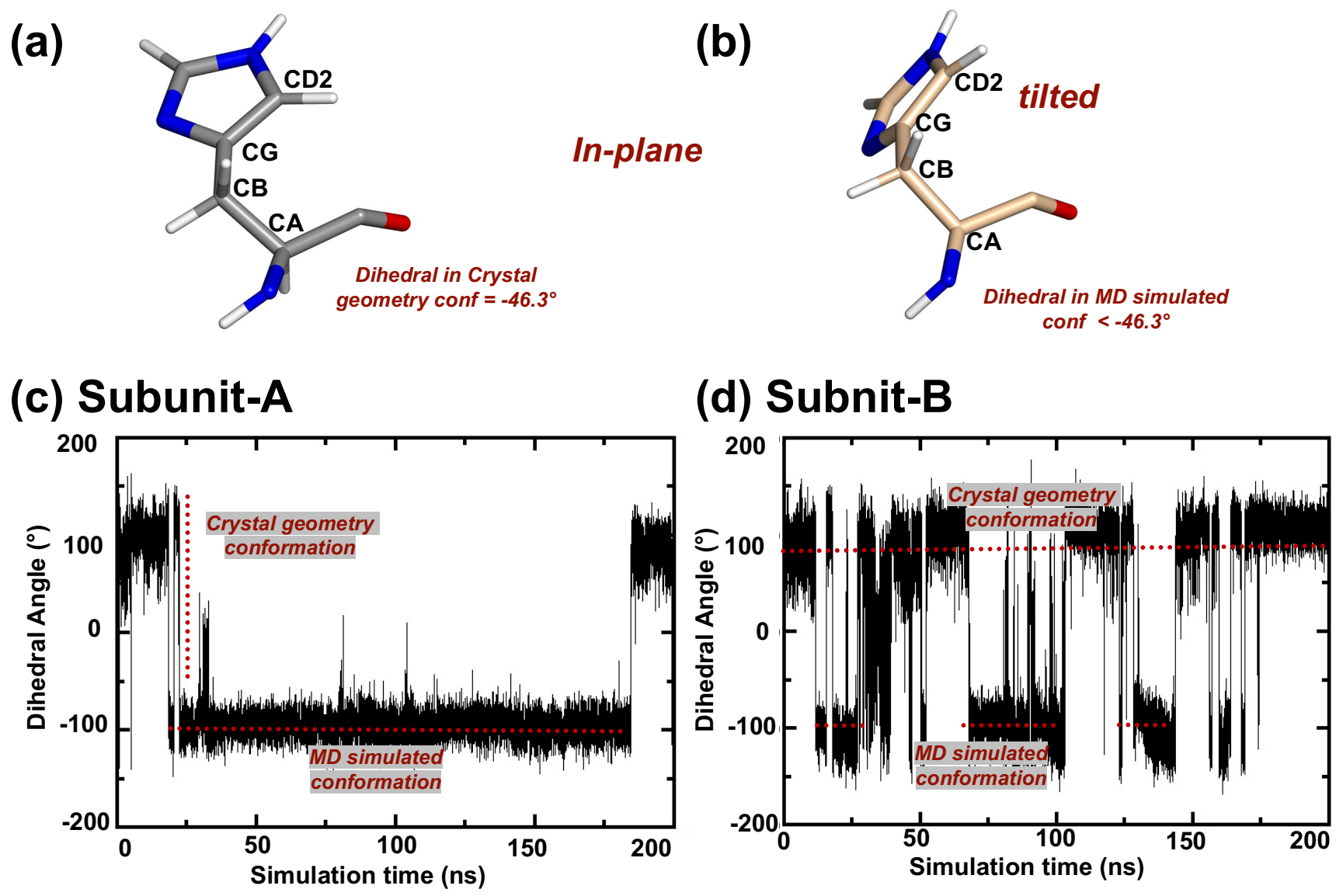

Figure S6. Dihedral distortion of His96 residue during MD simulation. (a) Crystallographic in-plane orientation of the His side chain, defined by the CA–CB–CG–CD2 dihedral angle. (b) MD-sampled tilted orientation of the same residue, showing a shifted CA–CB–CG–CD2 dihedral angle. (c) Dihedral-angle profile in subunit-A during the 200 ns MD simulation. (d) Dihedral-angle profile in subunit-B during the 200 ns MD simulation. The profiles indicate that the His96 side chain samples a tilted MD-favored conformation relative to the crystallographic orientation in both subunits.

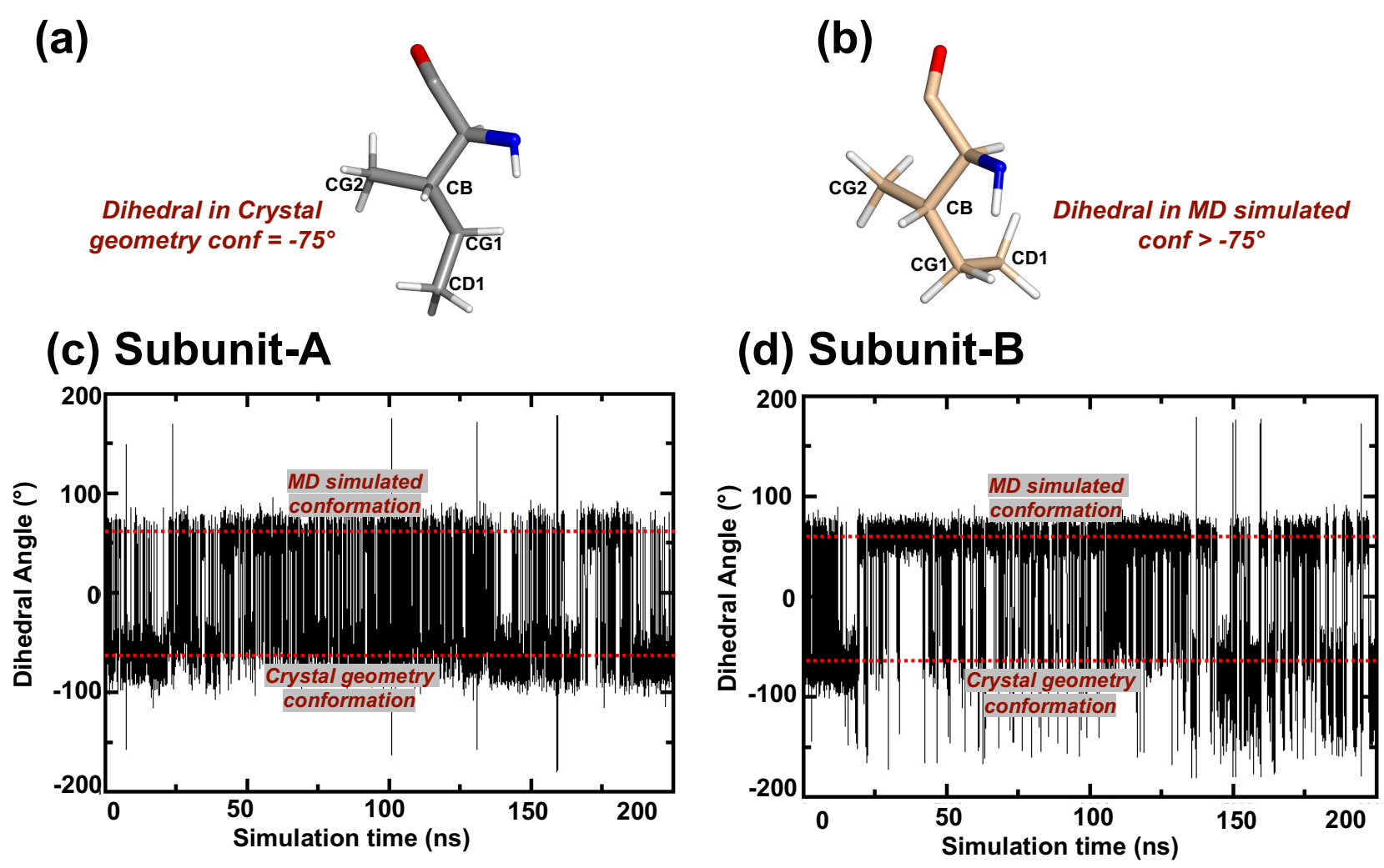

Figure S7. Dihedral distortion of Ile567 during MD simulation. (a) Crystallographic orientation of Ile567, corresponding to a CD1-CG1-CB-CG2 dihedral angle of approximately −75°. (b) MD-sampled orientation of Ile567, showing a shifted dihedral angle of approximately +75°. (c) Dihedral-angle profile of Ile567 in subunit-A during the 200 ns MD simulation. (d) Dihedral-angle profile of Ile567 in subunit-B during the 200 ns MD simulation. The profiles indicate that Ile567 samples an MD-favored rotated conformation in both subunits relative to the crystallographic geometry.

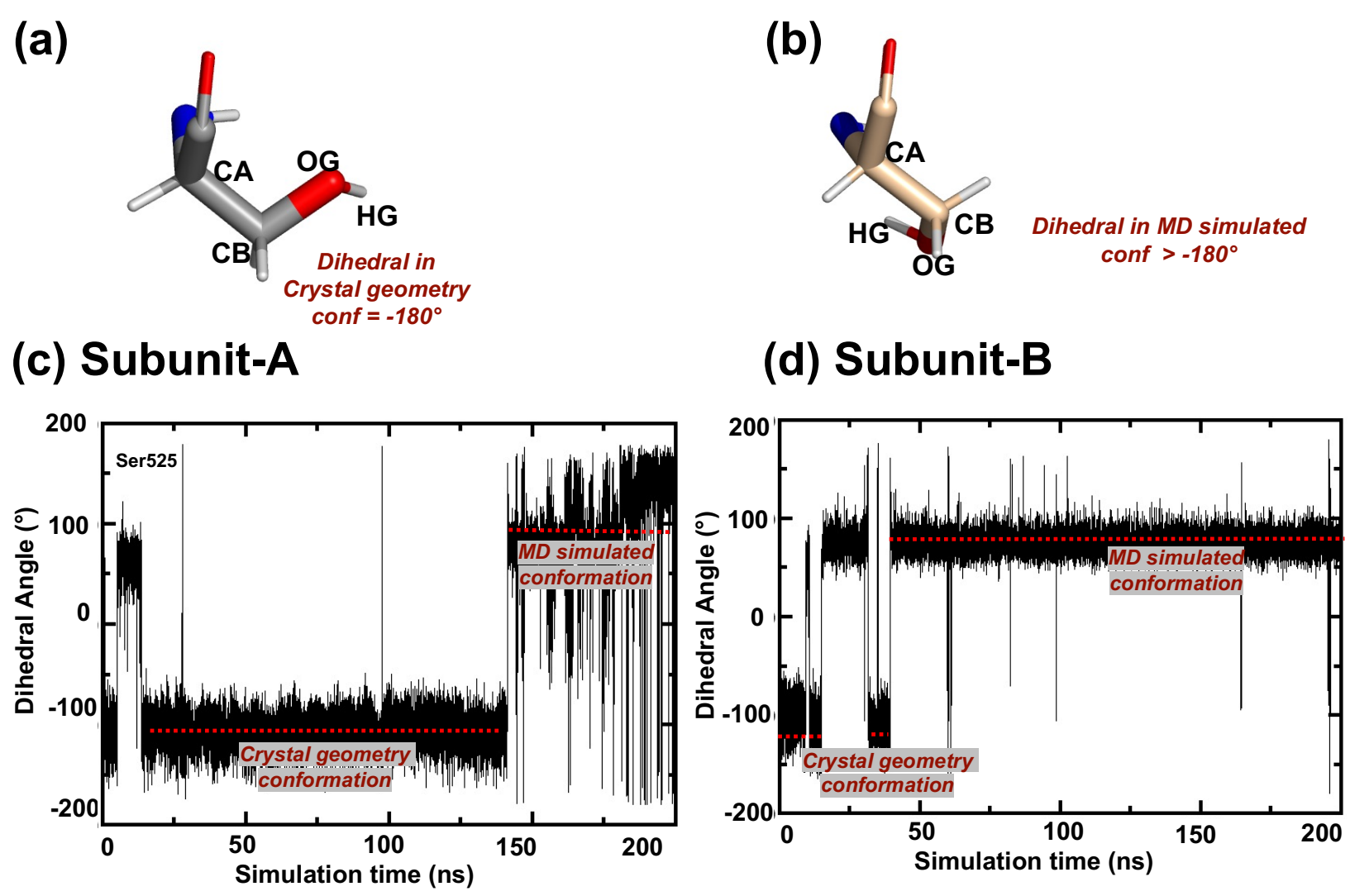

Figure S8. Dihedral distortion of Ser525 during MD simulation. (a) Crystallographic orientation of Ser525, corresponding to a CA-CB-OG-HG dihedral angle of approximately −180°. (b) MD-sampled orientation of Ser525, showing a shifted dihedral angle. (c) Dihedral-angle profile of Ser525 in subunit-A during the 200 ns MD simulation. (d) Dihedral-angle profile of Ser525 in subunit-B during the 200 ns MD simulation. The profiles show that Ser525 undergoes a transition from the crystallographic conformation to an MD-favored orientation in both subunits.

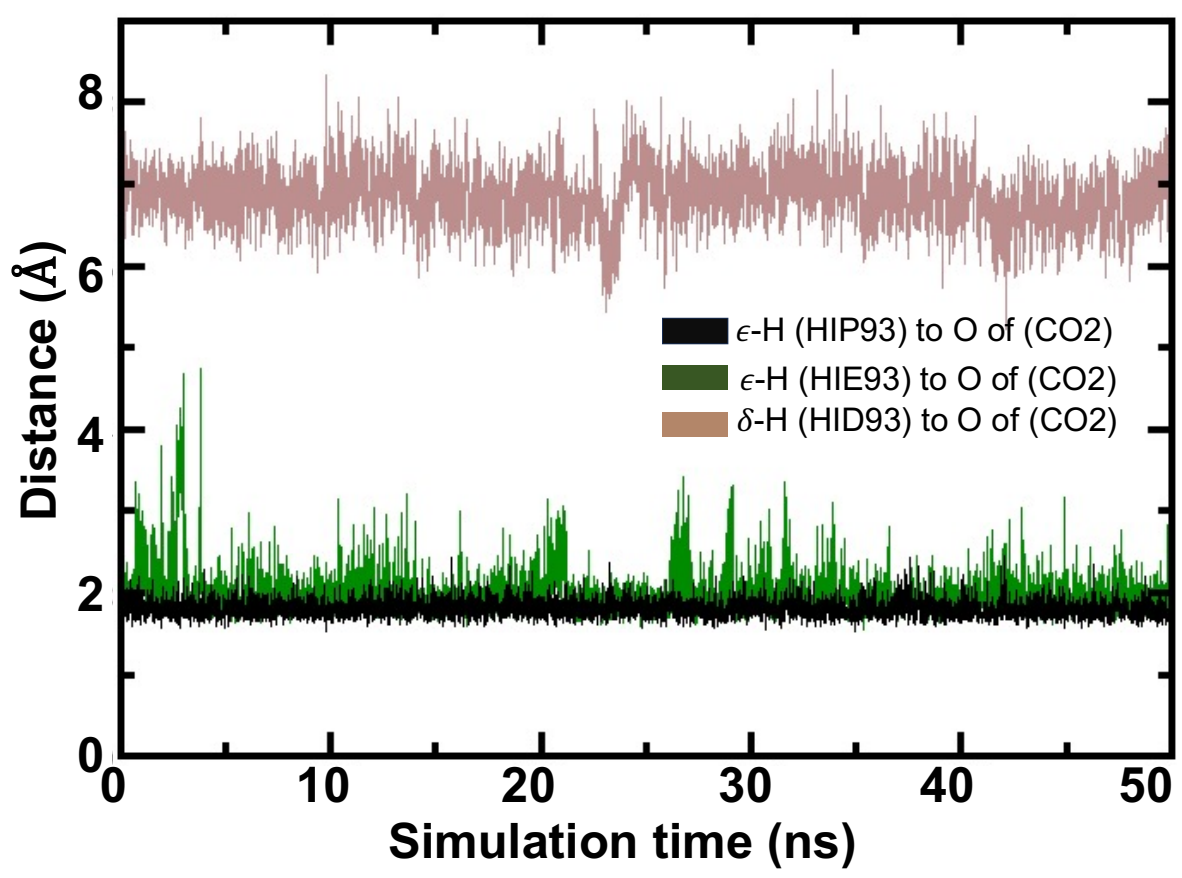

Figure S9. Interaction distance between nearest proton donor (His93) and proton acceptor CO_2.

References

1. C. L. Drennan, J. Y. Heo, M. D. Sintchak, E. Schreiter and P. W. Ludden, *Proc. Natl. Acad. Sci. U. S. A.*, 2001, **98**, 11973-11978.
2. H. Dobbek, V. Svetlitchnyi, J. Liss and O. Meyer, *J. Am. Chem. Soc.*, 2004, **126**, 5382-5387.
3. J.-H. Jeoung and H. Dobbek, *Science*, 2007, **318**, 1461-1464.
4. J. H. Jeoung and H. Dobbek, *J. Am. Chem. Soc.*, 2009, **131**, 9922-9923.
5. J. H. Jeoung and H. Dobbek, *J. Biol. Inorg. Chem.*, 2012, **17**, 167-173.
6. J. Fesseler, J. H. Jeoung and H. Dobbek, *Angew. Chem., Int. Ed.*, 2015, **54**, 8560-8564.
7. A. Ciaccafava, D. Tombolelli, L. Domnik, J. Fesseler, J. H. Jeoung, H. Dobbek, M. A. Mroginski, I. Zebger and P. Hildebrandt, *Chem. Sci.*, 2016, **7**, 3162-3171.
8. L. Domnik, M. Merrouch, S. Goetzl, J. H. Jeoung, C. Léger, S. Dementin, V. Fourmond and H. Dobbek, *Angew. Chem., Int. Ed.*, 2017, **56**, 15466-15469.
9. E. C. Wittenborn, M. Merrouch, C. Ueda, L. Fradale, C. Leger, V. Fourmond, M.-E. Pandelia, S. Dementin and C. L. Drennan, *Elife*, 2018, **7**, e39451.
10. E. C. Wittenborn, S. E. Cohen, M. Merrouch, C. Léger, V. Fourmond, S. Dementin and C. L. Drennan, *J. Biol. Chem.*, 2019, **294**, 13017-13026.
11. E. C. Wittenborn, C. Guendon, M. Merrouch, M. Benvenuti, V. Fourmond, C. Léger, C. L. Drennan and S. Dementin, *ACS Catal.*, 2020, **10**, 7328-7335.
12. M. Benvenuti, M. Meneghello, C. Guendon, A. Jacq-Bailly, J. H. Jeoung, H. Dobbek, C. Léger, V. Fourmond and S. Dementin, *Biochim. Biophys. Acta, Bioenerg.*, 2020, **1861**, 148188.
13. O. N. Lemaire and T. Wagner, *Biochim Biophys Acta Bioenerg*, 2021, **1862**, 148330.
14. A. Biester, S. Dementin and C. L. Drennan, *J. Inorg. Biochem.*, 2022, **230**, 111774.
15. J. H. Jeoung, J. Fesseler, L. Domnik, F. Klemke, M. Sinnreich, C. Teutloff and H. Dobbek, *Angew. Chem., Int. Ed.*, 2022, **61**, e202117000.
16. Y. Basak, J.-H. Jeoung, L. Domnik, J. Ruickoldt and H. Dobbek, *ACS Catal.*, 2022, **12**, 12711-12719.
17. S. M. Kim, J. Lee, S. H. Kang, Y. Heo, H. J. Yoon, J. S. Hahn, H. H. Lee and Y. H. Kim, *Nat. Catal.*, 2022, **5**, 807-817.
18. S. M. Kim, S. H. Kang, J. Lee, Y. Heo, E. G. Poloniataki, J. Kang, H. J. Yoon, S. Y. Kong, Y. Yun, H. Kim, J. Ryu, H. H. Lee and Y. H. Kim, *Nat. Commun.*, 2024, **15**, 2732.
19. O. D. Monera, T. J. Sereda, N. E. Zhou, C. M. Kay and R. S. Hodges, *J. Pept. Sci.*, 1995, **1**, 319-329.
